# REAPR: a universal tool for genome assembly evaluation

**DOI:** 10.1186/gb-2013-14-5-r47

**Published:** 2013-05-27

**Authors:** Martin Hunt, Taisei Kikuchi, Mandy Sanders, Chris Newbold, Matthew Berriman, Thomas D Otto

**Affiliations:** 1 Parasite Genomics, Wellcome Trust Sanger Institute, Wellcome Trust Genome Campus, Cambridge, CB10 1SA, UK; 2 Division of Parasitology, Department of Infectious Diseases, Faculty of Medicine, University of Miyazaki, Miyazaki 889-1692, Japan; 3 Weatherall Institute of Molecular Medicine, University of Oxford, John Radcliffe Hospital, Oxford, OX3 9DS, UK

**Keywords:** Genome assembly, validation, evaluation

## Abstract

Methods to reliably assess the accuracy of genome sequence data are lacking. Currently completeness is only described qualitatively and mis-assemblies are overlooked. Here we present REAPR, a tool that precisely identifies errors in genome assemblies without the need for a reference sequence. We have validated REAPR on complete genomes or *de novo *assemblies from bacteria, malaria and *Caenorhabditis elegans*, and demonstrate that 86% and 82% of the human and mouse reference genomes are error-free, respectively. When applied to an ongoing genome project, REAPR provides corrected assembly statistics allowing the quantitative comparison of multiple assemblies. REAPR is available at http://www.sanger.ac.uk/resources/software/reapr/.

## Background

The volume of genome sequence data continues to increase exponentially yet methods that reliably assess the quality of assembled sequence are lacking. In an attempt to categorise the quality of genome assemblies, Chain *et al. *[1] proposed a series of qualitative descriptions. Although these serve as a useful guide, they do not provide statistical or numerical comparisons of data quality apart from the extreme case of a 'finished' sequence. The recent advent of so-called next generation sequencing (NGS) has seen a dramatic increase in the rate of production of new genome sequences, with a growing proportion of genome projects classified as 'permanent draft' [2]. Moreover, most published assemblies do not get classified but are in fact also of 'draft' quality [3], which is the least accurate of all the categories. Relatively few reference genomes undergo continuous and rigorous quality improvement to repair errors. Two notable exceptions are the human genome [4] and the *Plasmodium falciparum *genome [5], where versioned error correction allows the comparison of sequence improvements over time. The reliability of reference sequence data is crucial for the interpretation of downstream functional genomic analysis and thus a metric indicating the genome wide accuracy of the reference sequence is essential.

Over 35 different tools ('assemblers') are available to perform *de novo *genome assembly [6]. The assembly of the short reads produced by NGS technology is however known to be problematic [7,8], despite the high coverage and range of insert sizes available. The precise behaviour of assemblers on a given genome is hard to predict without prior knowledge of its base composition, size, repetitive sequences and levels of polymorphism. Often the solution is to run assemblies with multiple tools or parameters and pick the best one based on summary statistics. Frequently, contig or scaffold N50 sizes are reported (the contig/scaffold size above which half the genome is represented) but although these are supposed to indicate contiguity (and certainly not accuracy), the frequent inclusion of incorrectly joined sequences provides a false boost to N50s despite reducing the accuracy of the genome consensus sequence. A better approach is to make a more informed decision on the best assembly by considering the real contiguity together with the errors in each assembly. Recent assembler evaluations GAGE [9] and Assemblathon 1 [10] highlighted the variability in performance of assemblers when given different input data or when changing their parameters. However, studies such as these require a known reference genome in order to assess the assemblies - a luxury that is unavailable when producing a *de novo *assembly.

The development of genome assembly analysis tools that do not require the use of a reference sequence for comparison is currently an active area of research, with a few tools already available. All tools share the similarity that they use the position of read pairs within an assembly to perform their analysis. Amosvalidate [11] was developed before the introduction of NGS, requires a file format produced by few assemblers and does not scale well to the large volumes of data typified by modern genome projects. Subsequent tools were recently introduced to work with NGS, all of which analyse assemblies using remapped reads and are effective at determining the best assembly from a set of assemblies of the same data. CGAL [12] and ALE [13] both produce a summary likelihood score of an assembly, with ALE also reporting four likelihood scores for each base. FRCbam [14] uses many metrics to identify 'features', which correspond to erroneous regions of an assembly and are used to plot a feature response curve [15]. The best assembly can be determined by overlaying these curves.

However, all of these tools lack the crucial ability to transform metrics into accurate error calls, or to report a single score for each base that defines whether the assembly is correct or wrong at any given position. Therefore we developed a reference-free algorithm (REAPR - Recognition of Errors in Assemblies using Paired Reads), applicable to large genomes and NGS data, with two principle aims: to score every base for accuracy and to automatically pinpoint mis-assemblies. The output is aimed to be as useful and informative as possible to the end-user and includes the bases identified as 'error-free' (see later for a definition), the location of assembly errors, and a new assembly that has been broken at points of assembly error. This information allows the N50 to be recalculated into the *corrected N50 *metric, similarly to previous studies that required a reference sequence [9,10]. Thus, the combination of the number of error-free bases and the corrected N50 can now provide an effective summary of any genome assembly.

## Results and discussion

### Overview of the REAPR pipeline

**Figure 1:**
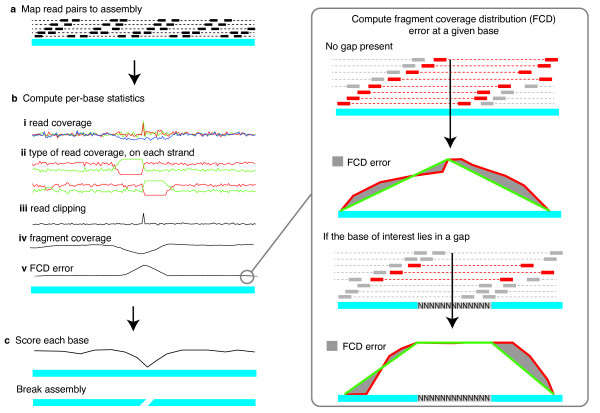
**Overview of the REAPR pipeline**. (**a**) The input is a BAM file of read pairs mapped to the assembly. (**b**) Statistics are calculated at each base of the genome: (**i**) Read coverage per strand, and any perfect and uniquely mapped read coverage is incorporated; (**ii**) The type of read coverage on the forward (upper plots) and reverse (lower plots) strand: proportion of reads that are properly paired (red), orphaned (green), and in the wrong orientation or exceed the fragment size range (not shown); (**iii**) The number of reads soft-clipped at each base; (**iv**) The fragment coverage, determined by the properly paired reads; (**v**) FCD error, taking into account the presence of a gap. Boxed are: FCD calculation at a given base. The fragments covering that base, shown in red, are used to construct a fragment depth plot (red). The FCD error is the area (grey) between the observed (red) plot and ideal plot (green). Since no read can map to a gap in the assembly, the calculation is corrected when a gap is present. (**c**) The statistics at each base are used independently to assign a score to each base of the assembly and also to break the assembly at scaffolding errors.

The REAPR pipeline uses the inherent information contained within sequencing reads mapped to an assembly (Figure 1, Additional file 1, Figure S1). Size-selected DNA fragments are typically sequenced from either end, resulting in paired reads separated by a space determined by the fragment size and sequencing technology. Our algorithm uses mapped paired-end reads to test each base of a genome sequence in two different ways. Small local errors (such as a single base substitutions, and short insertions or deletions) are detected within the mapped reads themselves and structural errors (such as scaffolding errors) are located using changes to the expected distribution of inferred sequencing fragments.

### Base-by-base analysis

**Figure 2:**
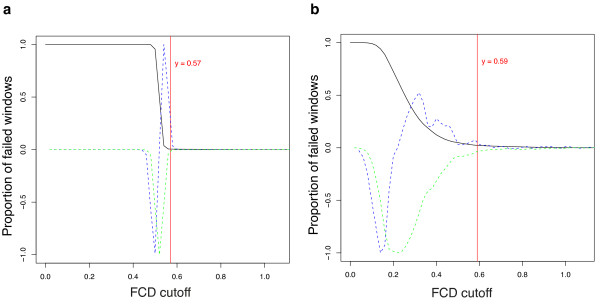
**Automatic calculation of the FCD error cutoff**. (**a**) *S. aureus de novo *assembly (*k*-mer of 71). (**b**) *P. falciparum de novo *assembly (*k-*mer of 55). In each plot, the black line shows the proportion of windows that would be called as an error for a range of cutoff values. The green and blue lines are the first and second derivatives of the black line, normalised to lie between -1 and 1. The vertical red line marks the FCD error cutoff, automatically determined by REAPR as the first FCD score corresponding to first and second derivatives ≥0.05.

A range of metrics, described in depth later, is extracted from the mapping information (Figure 1b) at each base of the genome assembly. Each read must be accurately mapped independently of its mate, so that a read pair is not artificially forced to map as a proper pair (in the correct orientation and separated by the correct distance, determined by the library type), otherwise the sensitivity in identifying assembly errors is reduced. The most important metric is derived from an analysis of fragment coverage, where a fragment is defined to be the region of the genome between the outermost ends of a proper read pair (Additional file 1, Figure S2). At a given base of the assembly, REAPR constructs a plot called the fragment coverage distribution (FCD) of the fragment depth arising from only the fragments that are mapped to that base (Figure 1b(v)). The difference between the theoretical and observed FCD, called the FCD error, is measured by taking the area between the two plots. REAPR uses the per-base FCD error to pinpoint assembly errors by reporting regions of the assembly containing a run of high FCD errors. The cutoff in FCD error, above which a base is called as incorrect, is automatically determined by sampling windows in the genome to determine how many windows fail at a range of cutoff values (Figure 2). The idea is to capture the plot's turning point, to the left of which the majority of windows fail due to background noise (see online Methods for a complete explanation).

Since a read cannot map to a sequencing gap (a region of ambiguous bases, or Ns), the theoretical FCD changes in the presence of a gap and a correction is applied to the FCD error calculation (Figure 1b(v), Additional file 1, Section 2.3), enabling the identification of scaffolding errors. In this way, REAPR scans along the entire genome, constructing the FCD at each base (Additional file 2), calculating the FCD error and identifying mis-assemblies.

In order to measure local accuracy REAPR uses proper read pairs that map to just one position of the assembly, with their entire length matching perfectly, to generate the read depth at every base of the assembly. By default, a given base is designated as locally error-free if it has at least five such reads aligned to it, but this is a parameter that can be changed by the user.

REAPR keeps track of several other metrics at every base of the genome. In terms of mis-assemblies, the most important of these is the fragment coverage where a value of zero returns an error. If it is non-zero then the value of the FCD error is taken into account. Any region that has no fragment depth, or has fragment distribution around a base that causes an FCD error, is reported as a mis-assembly. If this region contains a gap then it is likely to have arisen because two contigs have been falsely joined by read pairs that we term a scaffolding error, otherwise it is a simply an error in the assembled block of sequence that we term a contig error. In short, an assembly error call is triggered by either a lack of - or irregular - fragment coverage.

REAPR also outputs a warning for each of the following types of less serious inconsistencies in the assembly. A small deletion or insertion error often causes reads to be 'soft-clipped' (that is, some terminal bases ignored) in order for them to align to the assembly at the position of the error (see Additional file 1, Figure S2). Regions within an assembly where reads mapped in the wrong orientation, or as singletons, can aid in accurately determining the position of an FCD error caused by a scaffolding error or an incorrect assembly of a repetitive sequence. The latter pose a major challenge to assemblers, often resulting in collapsed repeats assembled into fewer copies than exist in the real genome. A region is flagged as a repeat by REAPR if the observed coverage is more than twice the expected coverage, after correcting for any GC bias present in the reads mapped to the assembly (Additional file 1 Figure S3d).

### Scoring each base of the assembly

REAPR assigns a score to every base of the assembly, with priority given to the perfect and unique read-pair coverage and the FCD error over other metrics. A given base is considered to be error-free, scoring one, if its FCD error is sufficiently small (see online Methods) and it is locally error-free (based on perfectly and uniquely mapped read depth, as defined above). This combination captures both the local accuracy and the presence of larger scale errors in an assembly, so that error-free bases represent the regions of the assembly that are extremely likely to be correct. Otherwise a score from zero to one is assigned, based on the number of other metrics that fall outside acceptable limits, with zero being the worst score. Briefly, the metrics used are the read depth and type of paired mapping, such as orphaned reads or reads in the wrong orientation, fragment depth and the presence of soft clipping (see online Methods for full details).

### Analysis of reference genomes

In order to evaluate the ability of REAPR to score each base of a genome and deduce the number of error-free bases, we applied it to two manually curated genomes of different isolates of *Staphylococcus aureus *(TW20 [16] and that of the GAGE dataset [9]) and to the *Plasmodium falciparum *genome, with its extreme base composition of only 19% GC (Table 1, Additional file 1 Tables S1-3). Both *S. aureus *reference genomes were found to be 98% correct (that is, 98% of bases were scored 1 by REAPR). Of the remaining 2% of bases, 96% fall within repeats. For *P. falciparum*, two successive public releases of the *P. falciparum *genome were analysed, with 94.4% error-free bases called in v2.1.4 and 94.9% in v3. We verified that REAPR correctly identified the changes that had been incorporated into the later version of the *P. falciparum *genome (Additional file 1 Table S4). These comprised a rearrangement between chromosomes 7 and 8 and a deletion in chromosome 13 and have been independently discovered using an optical map of the genome [17]. The corresponding breakpoints were all flagged by REAPR in version 2.1.4 of the genome. Further to the known errors in the *P. falciparum *genome sequence, four new collapsed repeats were discovered by REAPR (Additional file 1, Table S5). One of these collapsed repeats contains a gene previously reported to have a different copy number from that of the reference genome [18] (fully discussed in Additional file 1). Correcting another one of these regions resulted in the discovery of two new members of the *var *gene family (Additional file 1, Figure S4), an important and extensively studied family involved in malaria pathogenesis [19]. This error and the deletion in chromosome 13 were not detected during the significant amount of manual finishing work undertaken on the genome.

**Table 1:** A summary of REAPR results on a range of genome sequences.

						Scaffold errors^a^			
							
Genome assembly	Total length (Mb)	Gaps (*n*)	Total gap length (bp)	Original N50 (Mb)	Corrected N50^b^ (Mb)	Called by REAPR	False +ve	False -ve	Error-free bases (%)
*S. aureus *TW20 k71	3.0	31	249	0.2	0.2	18	2	0	98.2
*S. aureus*, GAGE Velvet	2.9	128	17,688	0.8	0.2	24	0	1	89.5
*P. falciparum de novo *k55	23.8	11,636	2,638,349	0.4	0.3	56	1	8	81.2
*P. falciparum *v2.1.4	23.3	160	947	1.7	1.7	4	1	0	94.5
*P. falciparum *v3	23.3	0	0	1.7	1.7	NA	NA	NA	94.9
*C. elegans *WS228	100.3	0	0	17.5	17.5	NA	NA	NA	90.3
*M. musculus *GRCm38	2725.5	522	77,999,939	130.7	100.2	41	ND	ND	80.1
*H. sapiens *GRCh37	3095.7	360	234,350,278	155.3	146.4	6	ND	ND	79.1

^a^Scaffold errors are not applicable (NA) when the assembly contains no gaps. Where a second genome sequence was unavailable for comparison, false-positives and false-negatives were not determined (ND).

^b^Corrected N50 refers to the N50 of the assembly after breaking the original assembly at breakpoints called by REAPR.

Next we applied REAPR to the *C. elegans *reference genome using a large insert size library that was derived from whole genome amplified (WGA) DNA. Ninety percent of the genome was reported to be error-free. The FCD error metric flagged up 842 errors, with manual analysis revealing that many of these error calls were caused by extremely uneven coverage across the genome. This unevenness was presumably a result of the WGA step used in the sequencing protocol (Additional file 1, Figure S5). However, the 20 regions with the largest FCD error were chosen for further analysis by PCR (Additional file 1, Figure S6, Table S6). Of the eight loci we were able to amplify, seven had a different size (>1.5 kb) from that predicted by the reference genome. Therefore REAPR successfully identified these regions as incorrect in the reference genome.

REAPR also scales to the human and mouse genomes, requiring less memory and CPU time than that of the mapping step (Additional file 1, Table S7). Ignoring sequencing gaps, we found 86% and 82% of bases to be error free, in the reference genomes of *H. sapiens *and *M. musculus*, respectively.

### Application to *de novo *assemblies

**Figure 3:**
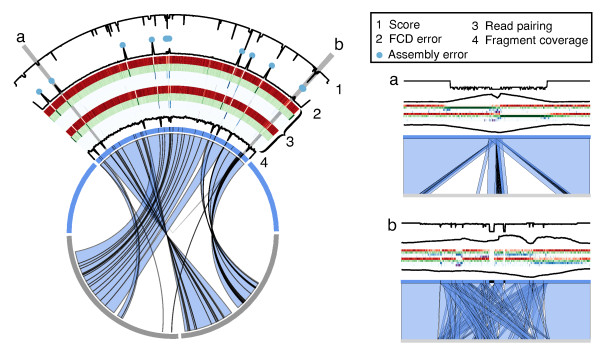
**Visualization of REAPR output after analysing a *de novo *assembly**. The results of running on the GAGE *S. aureus *Velvet assembly are shown (partly displayed using Circos [36]). Similarity by BLAST between the reference (grey) and the assembly (blue) is marked with blue bands. Only the BLAST hits to the largest scaffold from the assembly are shown, representing approximately 30% of the genome. One megabase of reference sequence that does not match the assembly supercontig of interest has been removed. The top plot shows the score output from REAPR, with the highest values corresponding to error-free bases. Error calls are marked with blue circles. The second plot shows the FCD error, with peaks corresponding to the error calls and low score regions. Next, heatmaps of the type of read coverage are shown for the forward and reverse strand: proper read pairs (red), orphaned reads (green), reads mapped too close or too far apart (blue) and reads oriented incorrectly (purple). The bottom plot shows the fragment coverage. (**a**, **b**) show zoomed in regions of the figure. (a) A deletion from the assembly, where the score drops, the FCD error increases and most reads flanking the deletion are orphaned. (b) A region of the assembly containing many repeats.

To test the supposition that REAPR should be able to find most types of assembly errors, first we applied it to the *S. aureus *dataset used in the GAGE paper, which contains several *de novo *assemblies and a comparison of each against the reference [9]. REAPR was run on all assemblies (Additional file 1, Tables S1-3), and the assembly containing the most errors was analysed in depth by manually comparing the GAGE assembly with the reference sequence using ACT [20] (Figure 3). REAPR correctly identified all 24 scaffolding errors in the assembly, with no false-positives (Additional file 1, Table S8). Next, we applied REAPR to *de novo *assemblies of the *S. aureus *genome. In each case, the availability of high quality reference genomes, with a reasonably small size, meant that we could validate error calls by manual comparison of the *de novo *assembled and reference sequences using ACT. We produced several assemblies of *S. aureus*, using a range of *k-*mer lengths. Manual inspection of the *k*=71 *de novo *assembly of *S. aureus*, showed that REAPR identified all 16 scaffolding errors, with only two false-positives (Additional file 1, Table S9).

We finally tested REAPR's applicability to a more challenging genome project by applying it to a *de novo *assembly of *P. falciparum*, which contained 11,636 sequencing gaps. In this case 55 scaffolding errors, again manually verified, were correctly identified with only one false-positive reported (Additional file 1, Table S10).

It should be noted that the ability of REAPR to detect errors is inherently limited by aspects of the sequencing technology such as insert size and read length meaning that some assembly errors remain unreported (see Additional file 1 for a full explanation). Further it should also be noted that assemblies of diploid (or polyploid) genomes still present a considerable challenge. Depending on the divergence between haplotypes, sequences may assemble separately or merge together. REAPR will call errors at the boundaries of regions where sequence-coverage differs, such as the boundary between merged and separated haplotypes. However, fully testing this functionality remains an area for future development alongside the development of assembly technologies that allow the sequences of homologous chromosomes to be assembled independently.

### Corrected assembly statistics

**Figure 4:**
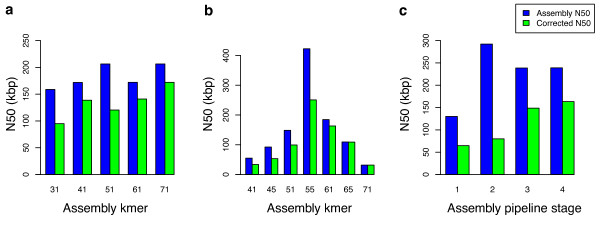
**N50 statistics of various assemblies before and after correcting with REAPR**. Blue bars show the N50 of the assembly input to REAPR, green bars show the corrected N50. (**a**) *De novo *assemblies of *S. aureus*. (**b**) *P. falciparum de novo *assemblies. (**c**) *B. pahangi *assemblies at four different stages of the genome project (see Additional file 1 for details).

The accuracy of REAPR allows the specific position of an error to be located in a scaffold. Using this information, scaffolds can be automatically broken wherever a scaffolding error occurs and contiguity statistics (N50, and so on) can be recalculated for the new improved assembly, thus providing a more accurate description of assembly contiguity (Figure 4, Additional file 1 Table S1). For example, although the original N50 of the *k*=51 and *k*=71 *S. aureus *assemblies were nearly identical at 206 kb, REAPR showed that the *k*=71 assembly was in fact significantly better with a corrected N50 of 172 kb, compared to 120 kb for the *k*=51 assembly. The resulting improved assembly, although more fragmented than the original, will be a better representation of the real genome sequence. For the *P. falciparum *assemblies, *k*=55 gives the best corrected N50, however larger values of *k *give more fragmented but also more accurate assemblies (Figure 4b).

Therefore, when applied to each of a series of *de novo *assemblies, REAPR arms the user with a robust method of comparing the output of different assemblers, so that the best assembly can be chosen for publication using standard but corrected metrics. To demonstrate this we applied REAPR to an ongoing genome project on the nematode *Brugia pahangi*. Figure 4c compares the progress of the assembly when monitored by standard N50 and REAPR corrected statistics at different steps of the improvement pipeline. Although the N50 itself does not increase at each stage, the corrected N50 shows a consistent increase and we see that genuine improvements have been made to the assembly.

## Conclusions

Here we have described the first algorithm that translates per-base metrics into error calls of reference sequences and *de novo *assemblies using NGS data. Establishing the quality of those sequences will become increasingly important as the assembly process shifts to more automated methods [3]. For example, REAPR correctly identified the ALLPATHS assembly to be the best of the GAGE *S. aureus *assemblies, without using a reference sequence. This assembly had the fewest error calls, the greatest number of error-free bases and the fewest warnings reported by REAPR (Additional file 1, Tables S1-3). Therefore we propose that REAPR should be applied to all genome projects prior to computing standard contiguity statistics (such as the N50). In this way the quality of assemblies and performance of assemblers can be compared robustly via a method that produces metrics that are constant between methodologies or datasets. By also providing a per base value for the accuracy of a sequence, that can be easily overlaid and viewed by the end-user, different genomes or assembly versions can be accurately compared and downstream analysis enhanced by enabling the end-user to be aware of regions of questionable accuracy.

## Materials and methods

### Read mapping

The read mapper SMALT [21] was used in all examples to map sequencing reads to assemblies. The entire command lines used are given in Additional file 1, but we note that the -x option was always used, so that each read in a mate pair was independently mapped thereby avoiding the false placement of a read near to its mate, instead of elsewhere with a better alignment. The -r option was also always used to randomly place reads which map repetitively, to prevent all repetitive regions of the reference sequence from having zero read coverage. After mapping, duplicate read-pairs were marked using the MarkDuplicates function of Picard version 1.47 [22].

### REAPR pipeline

The assembly analysis algorithm was implemented in a tool called REAPR: 'recognition of errors in assembly using paired reads'. The pipeline is simple to run, requiring as input an assembly in FASTA format and read pairs in FASTQ format. Alternatively, the user can map the reads to the assembly and provide a BAM file [23]. The steps in the pipeline are outlined in Figure 1 and described below (see Additional file 1 for full details of each stage).

Initially, input to the REAPR pipeline must be generated, starting with the unique and perfectly aligned read coverage of a high quality set of paired reads. For small genomes (<100 MB), this is calculated using the extremely fast but high memory tool SNP-o-matic [24]. For large genomes, the coverage is extracted from a BAM file of reads mapped using SMALT. This perfect and unique mapping information, together with a BAM file of the larger insert size reads mapped to the genome, is used as input to the REAPR pipeline. REAPR version 1.0.11 was used in all cases, with the default parameters.

The pipeline begins with a pre-processing step that estimates various statistics, such as average fragment length and depth of coverage, using a sample of the genome. In particular, GC bias is accounted for by calculating the expected fragment coverage at any given value of GC content. This correction to the fragment coverage is applied in subsequent stages of the pipeline. The method used is to take a LOWESS line through a scatter plot of fragment coverage *versus *GC content (see Additional file 1, Figure S3d).

The next stage calculates statistics at each base of the assembly, using the information in the input BAM file and the perfect and uniquely mapped read depth. These statistics are used to call errors in the assembly and to score each base of the assembly. We shall use 'inner fragment' to mean the inner mate pair distance or, equivalently, a fragment without including the reads (see Additional file 1 Figure S2a). The metrics calculated are read depth and type of read coverage, inner fragment coverage, error in inner fragment coverage (corrected for GC content), FCD error and amount of soft clipping. The metrics are explained in more detail below and in Additional file 1.

Recall that the FCD error at each base of an assembly is taken to be the area between the observed and ideal fragment coverage distributions (see Figure 1c). It is normalized for both fragment depth and mean insert size so that results are comparable for data from different libraries. A correction is made for the presence of the nearest gap, if it lies within one insert size of the base of interest (see Additional file 1). If a base has zero fragment coverage then this metric cannot be used and the assumption is that the assembly is incorrect. The exception to this is where a gap has length longer than half the average insert size, in which case it is impossible to determine if this scaffolding is correct and therefore no further analysis is performed.

In addition to the absolute count of read coverage, the type of read coverage is considered. At each base, and for each strand, the proportion of reads of the following types is calculated: proper read pairs, defined to be in the correct orientation and insert size, which should be in the majority if the genome is correct; orphaned reads, whereby a read's mate is either unmapped or mapped to a different chromosome; reads with the correct orientation but wrong insert size; and read pairs with an incorrect orientation.

Most read mapping tools are capable of soft-clipping reads, where most of a read is aligned to the genome, but a few bases at either end of the read do not match. In this case the read is still reported as mapped, but the mismatching bases are not considered as part of the alignment and designated as soft-clipped (Additional file 1, Figure S2c). At each base, the number of alignments is counted that start or end at that base due to a soft-clipped read.

In order to call assembly errors from a given metric, a minimum window length is considered and appropriate minimum and maximum values. Any region of length no smaller than the window length and with at least 80% of the bases falling outside the acceptable range is reported. For example, a collapsed repeat is called if the relative error in fragment coverage is at least two for 80% of the bases in a stretch of at least 100bp. The default choice of parameter for each metric is described in the Additional file 1. In the actual implementation, the user can choose all parameters.

As described earlier, each base scores one if it is covered by at least five perfect and uniquely mapped reads, and the FCD error is acceptable. If either of these tests fail, then the score is set to the number of tests that pass (considering all per-base metrics) scaled from zero to one, that is, a base scores zero if every test fails. The FCD error cutoff is chosen by sampling windows from the genome, then for each window the cutoff in FCD error needed to call that window as an error is calculated. In other words, for each window we find the value *c *such that 80% of the values in that window are greater than *c*. The proportion of failed windows as a function of cutoff value is plotted (Figure 2). The cutoff value for the FCD error is chosen to be the first value found, working from largest to smallest, such that the magnitude of the first and second derivatives (normalized to have a maximum magnitude of 1) of the plot are both at least 0.05.

### REAPR output

REAPR reports assembly errors and warnings in a GFF file, compatible with most genome viewers such as Artemis [25]. Regions with a high FCD error or low fragment coverage are reported as an error, whereas regions that fail any other tests are output as warnings for manual inspection. A summary spreadsheet is produced containing error counts, broken down in to each type of error, for each contig and for the whole assembly. REAPR also produces a new assembly based on the error calls by breaking the genome wherever an error is called over a gap. Error regions within contigs are replaced with Ns, enabling them to be accurately reassembled locally by a gap closing tool [26,27]. A second run of REAPR can be performed after gap closing to verify any new sequenced added to the assembly. REAPR also generates plot files, compatible with Artemis, of all the statistics examined at each base for easy visualisation (see Additional file 1, Figure S7 for an example).

### *De novo *assemblies

The *de novo *assemblies of *S. aureus *and *P. falciparum *were produced using similar methods (see Additional file 1 for full details). Short insert Illumina reads were assembled using Velvet [28] version 1.2.03. These assemblies were scaffolded iteratively with SSPACE [29] version 2 using the short insert reads, followed by further rounds of scaffolding with larger insert reads, where available.

### Assembly analysis

Manual comparison between the *de novo *assemblies and reference genomes of *S. aureus *and *P. falciparum *were performed using ACT [20]. BLAST hits between the sequences were generated for viewing in ACT using blastall version 2.2.15 with the settings -p blastn -W 25 -F T -m 8 -e 1e-20.

When counting scaffolding error calls in *S. aureus*, the Velvet assembly was found to contain three problematic regions, with many gaps and errors due to repetitive sequences. Each of these regions was counted as one scaffolding error for the purpose of calculating REAPR's performance at error calling.

The read sets used for *P. falciparum *assemblies were Illumina 500bp insert, Illumina 3 kb insert and 454 8 kb insert reads. The short insert Illumina reads were used to generate perfect and uniquely mapped read depth, and also to call collapsed repeats. All other errors were identified using the 454 reads.

Perfectly mapped and unique read depth was generated for the *C. elegans *genome (WS228) using three Illumina lanes combined and the larger insert size dataset comprised four combined Illumina lanes. Prior to mapping the latter reads, inner adaptor sequences were removed using in-house scripts based on SSAHA2 [30], retaining read pairs where each mate of the pair had a length of at least 35bp. PCR primers were designed to amplify the top 20 FCD error regions using AcePrimer 1.3 [31].

High coverage Illumina data [32] were used to analyse the human and mouse reference genomes. For each organism, the dataset comprised short insert data and more than one 2-3 kb insert 'jumping' library. The short insert data were used to compute the perfect and uniquely mapped read depth and the 2-3kb libraries were combined to obtain enough coverage for analysis with REAPR.

### Software

REAPR is open source and runs under Linux, with modest run time and memory requirements (Additional file 1, Table S7). It is written in C++ and Perl, relying on existing open source tools [23,33,34] and the BamTools C++ API [35]. A virtual machine is provided to enable Windows and Mac users to run REAPR.

### Data availability

The primary data for *Brugia pahangi *are available at the Short Reads Archive (SRA) under accession codes ERR070030 and ERR068352.

Other publicly available datasets used in this manuscript can be found in SRA under the accession codes: ERR142616 and SRR022868 (*S. aureus*); ERR034295, ERR163027-9 and ERR102953-4 (*P. falciparum*); ERR068453-6 and ERR103053-5 (*C. elegans*); SRR0676 (*M. musculus*); and SRR067577-9 and SRR0677 (*H. sapiens*).

## Abbreviations

Bp: base pair; FCD: Fragment Coverage Distribution; NGS: Next Generation Sequencing; REAPR: Recognition of Errors in Assemblies using Paired Reads.

## Competing interests

The authors declare that they have no competing interests.

## Authors' contributions

MH, TDO, MB and CN conceived the project and wrote the manuscript. The REAPR pipeline and assemblies were produced by MH. Assembly analysis was performed by MH and TDO. *C. elegans *and *P. falciparum *experimental work was carried out by TK and MS, respectively. All authors read and approved the final manuscript.

## Supplementary Material

Additional file 1 **Supplementary information**. Detailed methods, analysis and results to support the main text. Click here for file

Additional file 2 **Movie of the fragment coverage distribution over an assembly error**. Click here for file
