# Supplementary material for: REAPR: a universal tool for genome assembly evaluation
Source: Genome Biol. 2013 May 27;14(5):R47. doi: 10.1186/gb-2013-14-5-r47 (PMC3798757; doi:10.1186/gb-2013-14-5-r47)
Supplement: Additional file 1 — Supplementary information. Detailed methods, analysis and results to support the main text. [file gb-2013-14-5-r47-S1.PDF]

Supplementary Material of:

REAPR: a universal tool for genome assembly evaluation

Martin Hunt[1], Taisei Kikuchi[2,1], Mandy Sanders[1], Chris Newbold[3,1], Matthew Berriman[1], Thomas D Otto[1]

[1]Parasite Genomics, Wellcome Trust Sanger Institute, Wellcome Trust Genome Campus, Cambridge, CB10 1SA, UK

[2]Division of Parasitology, Department of Infectious Diseases, Faculty of Medicine, University of Miyazaki, Miyazaki 889-1692, Japan

[3]Weatherall Institute of Molecular Medicine, University of Oxford, John Radcliffe Hospital, Oxford, OX3 9DS, UK

Contents

1 Glossary **2**

2 REAPR pipeline **2**

2.1 Perfect and uniquely mapped read coverage 2
2.2 Preprocessing 3
2.3 Generate per-base statistics 3
2.4 Score each base and locate mis-assemblies 6
2.5 Breaking the assembly 7
2.6 REAPR output 7

3 Application to assemblies and reference genomes **7**

3.1 *Staphylococcus aureus* 8
3.2 *Plasmodium falciparum* 9
3.3 *Caenorhabditis elegans* 10
3.4 *Mus musculus* 11
3.5 *Homo sapiens* 11
3.6 *Brugia pahangi* 12

4 Figures and tables **13**

1 Glossary

We begin with a glosary of the most common technical words used throughout the manuscript, to remove any ambiguity for the reader.

Fragment	A length of DNA sequence (expected to be up to a few kilobases in length), which may or may not include its reads (see inner and outer fragment). 'Insert' is also used interchangeably for 'fragment'.
Read	A sequence of DNA at the end of a fragment.
Read pair	Two reads that are from each end of a fragment.
Inner fragment	A fragment, not including the read pairs (Figure S2a)
Outer fragment	A fragment (including the reads).
Gap	A run of unknown bases, specifically a string of Ns of length at least one, in a genome assembly.
Soft-clipping	Where a read can be mapped to the reference sequence, but to do so some bases at either end must be excluded from the alignment. These bases are said to be 'soft-clipped'.
Read coverage	At a given base of an assembly, the number of reads which are mapped over that base. Synonymous with read depth.
Fragment coverage	As for read coverage, but fragments are considered instead of reads (Figure S2b).

We emphasize that we use 'fragment' in circumstances where it does not matter if the reads themselves are included. If this distinction is important, then we use 'inner fragment' or 'outer fragment' as necessary.

2 REAPR pipeline

A schematic of the implementation of REAPR is shown in Figure S1. Each step of the pipeline is described in detail below.

2.1 Perfect and uniquely mapped read coverage

Recall from the main text that two sets of paired end reads were used to anaylse each assembly. The first (high quality) set is used to generate the coverage of perfectly and uniquely mapped reads at each base of the genome. This was calculated using two different methods, depending on the size of the assembly. For small genomes, the function `perfectmap` was used, which is essentially a wrapper for SNP-o-matic, making it easy to run for the end user.

The method for small genomes is extremely fast, but requires a large amount of memory to run. Therefore, for large genomes we implemented an alternative method with a low memory footprint. A BAM file of mapped reads was generated using SMALT, which was then used as input to the `perfectfrombam` function of REAPR. Any read mapper that outputs an alignment score can be used at this stage, provided it outputs the score using the standard tag `AS` in each BAM record.

The BAM file is then filtered by excluding any read pair where either read of the pair has an alignment score or mapping score below thresholds chosen by the user. The alignment score cutoff ensures that the read is perfectly aligned. The mapping score cutoff will exclude reads that could map equally well to more than one location in the assembly. These thresholds will vary depending on the mapper used. In the case of SMALT the minimum mapping score was set to 4, since repetitive reads are allocated a score up to and including 3. SMALT gives a score of 1 to a match, which meant that we set the minimum alignment score to be the read length, so that only reads with a perfect alignment could pass the filtering stage.

2.2 Preprocessing

Samples of the genome and BAM file are used to estimate various statistics before the pipeline is run on the full data set, as described in the main text. More details are provided below for the estimation of fragment size and the method of correcting for bias in coverage for varying GC content.

Since the following method is applied to both inner and outer fragment size distributions, we shall refer to just 'fragment' to include both inner or outer fragments. The first two million fragments are used to estimate the fragment size distribution, so that the 'average' fragment size can be estimated. The average is taken to be the mode fragment size, within one standard deviation of the mean fragment size. This is to account for unusual distributions from some libraries. For example, a bimodal fragment size distribution commonly arises from a large fragment library (where the mode fragment size is very small and should not be considered to be the average size, an example is shown in Figure S8). The minimum, m, and maximum, M, fragment sizes are estimated by taking the 1st and 99th percentiles of the distribution respectively. A mapped read pair is considered to be proper if the reads are in the correct orientation and the fragment size lies inside the interval $[m, M]$.

The GC content and fragment coverage is calculated for the first million bases of the genome, excluding gaps. A window size equal to the average (as defined in the previous paragraph) inner fragment size is used when calculating the GC content around each base. The dependency of inner fragment coverage on GC content is determined by calculating a LOWESS line through a scatter plot of these statistics using the `lowess()` function in `R` (Figure S3d). Any missing values are linearly interpolated, so that the expected coverage is known for each GC content in the range zero to 100. Also, the mean fragment coverage is calculated from this sample of one million bases for use when calculating the FCD error (explained below).

2.3 Generate per-base statistics

The `stats` function of REAPR generates the following statistics at each base of the assembly, using information in the input BAM file and perfect and unique mapping information.

- Inner fragment coverage. We use inner fragment coverage because this more accurately calls breakpoints than outer fragment coverage (Figure S2b).
- Relative error in inner fragment coverage, taking the expected coverage at a base using the LOWESS line described above.

- Read depth, broken down into forwards and reverse strands.
- The type of read coverage, broken down into forwards and reverse strands. The types are (a) proper read pairs (correct orientation and fragment length), (b) orphaned reads (mate either unmapped or mapped to a different contig), (c) reads with the correct orientation but wrong fragment size and (d) reads with an incorrect orientation.
- Perfect uniquely mapped read coverage, calculated using SNP-o-matic (for small genomes) or from a BAM file of reads mapped using SMALT (for large genomes).
- The number of reads soft-clipped, broken down into left or right end clipped and forward or reverse strand.
- The error in outer fragment coverage distribution (FCD error), explained in next section.

The outer fragment coverage distribution (FCD) is analysed at a given base of the assembly by considering all outer fragments covering that position (see Figure 1). The FCD plot is constructed at each base of the genome - Additional file 1 shows an example for the *C. elegans* genome in a region where an FCD error was correctly called. Deviation from the theoretical shape of this plot is measured by taking the area between the observed and expected values. We refer to this area as the *FCD error* for that particular base. A correction is applied to the expected values to account for the nearest gap (if the gap lies within a fragment length of the base under consideration).

We use the following notation:

$$\begin{aligned} i &= \text{average outer fragment size} \\ s &= \text{start position of gap} \\ e &= \text{end position of gap.} \end{aligned}$$

When calculating the theoretical plot heights, we normalise the expected depth by dividing all values by the total number of fragments covering the position of interest, so that the ideal plot intercepts the y axis at zero. For simplicity, we also shift the assembly coordinates in the following by assuming that the position of interest is zero.

The FCD error is calculated using the formula

$$\frac{1}{i} \sum_{n=\lfloor -3i/2 \rfloor}^{\lceil 3i/2 \rceil} \left| t_n - \frac{o_n}{o_0} \right|$$

where t_n and o_n are the theoretical and observed heights of the plots respectively. Note that this calculation ignores regions further than $1.5i$ from the position of interest, in order to prevent outliers dominating the result by causing an artificially large FCD error. We normalise by the mean fragment size, so that the ideal plot always intercepts the x axis at $\pm i$ (except in the presence of a gap).

The various cases of gap positions and associated ideal plots are shown in Figure S9. We now show the equations of the ideal plot height for each case.

(a) *No gap*: Figure S9a. If there is no gap within a frament length of the position of interest, then y is defined by

$$y = \begin{cases} 0, & |x| \geq i \\ \frac{1}{i}(x+i), & -i < x \leq 0 \\ \frac{1}{i}(x-i), & 0 < x < i \end{cases}$$

(b) *Gap over centre*: Figure S9b. In this case, we have

$$-i \leq s \leq 0 \leq e \leq i$$

and we assume that $e - s < i$.

The equation of the line is then given by

$$y = \begin{cases} 0, & x \leq e-i \text{ or } x \geq s+i \\ \frac{1}{s+i-e}(x-e+i), & e-i < x < s \\ 1, & s \leq x \leq e \\ -\frac{1}{s+i-e}(x-s-i), & e < x < s+i \end{cases}$$

(c) *Gap near centre*: Figure S9c, where the case $0 < s < e < i$ is shown, that is, the gap lies to the right of zero. The alternative case is that the gap lies to the left of zero, so that we have $-i < s < e < 0$. In which case, we simply change the sign of s and e so that $0 < s < e < i$. Therefore we may assume that the gap lies to the right of zero. The formula for the theoretical plot is then given by

$$y = \begin{cases} 0, & |x| \geq i \\ \frac{1}{s+i-e}(x+i), & -i < x < s-i \\ \frac{s}{s+i-e}, & s-i \leq x \leq e-i \\ \frac{1}{s+i-e}x+1, & e-i < x \leq 0 \\ -\frac{1}{s+i-e}x+1, & 0 < x < s \\ 1-\frac{s}{s+i-e}, & s \leq x \leq e \\ -\frac{1}{s+i-e}(x-i) & e < x < i \end{cases}$$

(d) *Gap far away from centre*: Figure S9d. This case can be broken down into two more cases: we either have

$$0 < s < i < e \qquad (*)$$

or

$$s < -i < e < 0 \qquad (\#)$$

Assuming that (∗) holds, which is the situation shown in Figure S9d, the equation of the line is

$$y = \begin{cases} 0, & x \leq -i \text{ or } x \geq s \\ \frac{1}{s}(x+i), & -i < x < s-i \\ 1, & s-i \leq x \leq 0 \\ -\frac{1}{s}(x-s), & 0 < x < s \end{cases}$$

If instead (#) is true, then we obtain

$$y = \begin{cases} 0, & x \leq -e \text{ or } x \geq i \\ -\frac{1}{e}(x-e), & e < x < 0 \\ 1, & 0 \leq x \leq e+i \\ \frac{1}{e}(x-i), & e+i < x < i \end{cases}$$

2.4 Score each base and locate mis-assemblies

Each of the statistics calculated in the previous stage are used to score each base of the assembly and to call errors in the assembly. This is performed by the `score` function of REAPR. Each metric is analysed by picking a window of fixed length and finding those windows where at least 80% of the bases in that window fall outside a given range. Except where noted, the default window length is 100bp and the cutoffs are as follows. The minimum read and inner fragment coverage is 1. There must be a depth of at least 5 perfectly and uniquely mapped reads, with a window of 30bp. Repeats are called using the inner fragment coverage, corrected for GC content as described previously. At each base we consider the relative error in the observed fragment coverage corrected for the GC content around that base, using a cutoff of 2. This means a repeat is called when the fragment coverage is twice the expected value.

At a given base, a count is made of the number of reads which are mapped with either end of the alignment starting at that base due to soft-clipping. For example, in Figure S2c the count is 10 (the number of red reads). There are four possibilities: the read could map to either strand and could have the left or right end clipped (in Figure S2c the strand is not marked, but there are five reads clipped at their left end and the same number clipped at their right end). At a given base b, let

$$\begin{aligned} d_f &= \text{read depth on forwards strand} \\ d_r &= \text{read depth on reverse strand} \\ n_{fl} &= \text{number of reads mapped to the forwards strand, whose} \\ &\quad\ \text{alignment starts at } b \text{ due to soft-clipping (left end clipped)} \\ n_{rl} &= \text{same as } n_{fl}\text{, but on the reverse strand.} \end{aligned}$$

A soft-clipping warning is generated from reads clipped at their left end if $d_f, d_r > 0$ and we have

$$n_{fl}/d_f \geq 0.5 \qquad \text{and} \qquad n_{rl}/d_r \geq 0.5.$$

A warning is generated in an analogous manner by considering reads clipped at their right end.

The window size used to call FCD errors is the average outer insert size, if this is less than 1000bp, otherwise half the insert size is used. The FCD error cutoff is determined by the REAPR function `fcdrate`, which analyses a sample of the windows to determine the proportion of windows called as incorrect for a range of cutoff values (Figure 2). The cutoff is chosen to be the first value found, considering the largest cutoff first and proceeding in decreasing order, such that the magnitude of the first and second derivative of the windows called as errors are both ≥ 0.05.

2.5 Breaking the assembly

The errors called are used to generate a new assembly, using REAPR `break`, in the following manner. Whenever an error is called over a gap, the gap is deleted and the assembly is broken at the gap position. Where an FCD or low fragment coverage error is called, that section is replaced with a gap, i.e. each base in that region is replaced with an N. If Illumina data are available, then a tool to fill gaps, such as IMAGE or GapFiller, can be subsequently run on the assembly.

2.6 REAPR output

REAPR outputs several plots, summarising various properties of the reads and the genome. Figure S3 shows the plots made by REAPR when run on *de novo* assembly of *S. aureus*. The errors and warnings are reported in a GFF file, which can be read by most genome viewers. An example line showing an FCD error is

```
Scaffold_1 REAPR FCD 85649 91352 0.520595 \
     . . Note="Error: FCD failure"
```

where (fields are tab-delimited and) the FCD error lies in

The `summary` function reports continuity statistics, such as the N50, of the original and broken assemblies, together with a breakdown of the number of errors and warnings produced. Finally, a utility is provided to make files suitable for viewing in Artemis for a given scaffold. An example can be seen in Figure S7, which shows an Artemis screenshot with all the plots generated by REAPR.

3 Application to assemblies and reference genomes

Version 1.0.11 of REAPR was used using the default settings in all cases. This entailed three steps:

- generate uniquely and perfectly mapped read coverage from a small insert library;
- generate a BAM file of reads from a large insert library mapped to the assembly;
- run the REAPR pipeline.

The details of these steps are given below for each organism, but the general calls for steps 1 and 3, except for the mouse and human genomes (see later for the details of these genomes), were the following.

```
reapr perfectmap assembly.fasta reads_1.fastq reads_2.fastq \
    insert_size perfect_prefix

reapr pipeline assembly.fasta in.bam output_directory \
    perfect_prefix
```

Here, `assembly.fasta` is the FASTA file of the assembly, the files `reads_1.fastq` and `reads_2.fastq` contain the forward and reverse reads, with an average fragment size of `insert_size`, and `perfect_prefix` is the prefix of the output files. `in.bam` is the BAM file of large insert reads mapped to the assembly.

All reads were mapped using SMALT, before marking duplicate read pairs with the `markDuplicates` function of Picard (`http://picard.sourceforge.net`). The resulting BAM file was used as input to REAPR.

3.1 *Staphylococcus aureus*

Two distinct datasets were used: data from strain TW20, used to generate and analyse *de novo* assemblies, and data from the GAGE website (`http://gage.cbcb.umd.edu/data/index.html`). The reference genome of TW20 was downloaded in the following files.
`ftp://ftp.sanger.ac.uk/pub/pathogens/sa/TW20_CDS.embl`
`ftp://ftp.sanger.ac.uk/pub/pathogens/sa/pTW20_1.embl`
`ftp://ftp.sanger.ac.uk/pub/pathogens/sa/pTW20_2.embl`
FASTQ files from strain TW20 (accession number ERR142616) were assembled using Velvet version 1.2.03, with k-mers 31, 41, 51, 61 and 71 when running `velveth`. The options `-ins_length 280 -cov_cutoff auto -exp_cov auto` were used in all assemblies when running `velvetg`. Each of these assemblies was scaffolded using SSPACE version 2 in an iterative manner, using the same Illumina reads that were assembled by Velvet. The minimum number of links needed was varied, using the `-k` option, with successive runs with values of 30, 20, 10, 10, 7, 7, 5, 5, 5. The minimum overlap required between contigs to merge adjacent contigs in a scaffold was set to 31, with the option `-n 31`. Reads were mapped to each assembly using SMALT version 0.6.2, with settings `-k 13 -s 2` when indexing the genome. `SMALT map` was run with the options `-x -r 1 -y 0.8 -i 1000`.

REAPR called two false-positive scaffolding errors and five contig errors in the *de novo* assembly with k-mer 71. All of these were caused by repetitive regions within the genome. An example is shown in Figure S10 that is typical of all the false-positive calls. Repeats caused the read pairs to map with a sub-population of significantly larger insert sizes, so that the FCD error was high enough to flag an error.

In order to analyse the GAGE *S. aureus* dataset, the assemblies and short jump reads were downloaded from the GAGE website. The short jump reads were mapped to each genome using SMALT version 0.6.2. The genome was indexed with `SMALT index -k 11 -s 2` and mapped with `SMALT map` using the options `-x -r 0 -y 0.7`

-j 2000 -i 5000. The short insert fragment reads were used to generate perfect and uniquely mapped read depth at each base, by running

```
reapr.pl perfectmap genome.scf.fa frag_1.fastq frag_2.fastq \
    165 perfect_out
```

on each genome. Instead of using the reads from the GAGE website, which were a subset of the full dataset, we used the original FASTQ files (accession number SRR022868). The REAPR pipeline was run on each genome using this information together with the mapped short jump reads using

```
reapr.pl pipeline genome.scf.fa short_jump.bam out_dir \
    perfect_out
```

Analysis of the comparison of the GAGE Velvet assembly with the reference genome, using the GAGE code, revealed that REAPR missed calling 18 small insertions or deletions (caused by repeats) as contig errors. 16 of these represented a length difference between the assembly and reference of not more than 100bp, with the remaining two less than 200bp. These were not flagged as FCD errors because the sensitivity was too low when using reads with an insert size of 3.4kb. Eight of the regions containing these errors were not called with error-free bases by REAPR, being assigned a score less than 1, showing that the regions could not be trusted. The remaining 10 regions had reads mapped perfectly and uniquely across them, which was possible because of the nature of the repetitive sequence. An example is shown in Figure S11. This type of mis-assembly error did not occur in the *de novo* assembly with a k-mer of 71, in contrast to the GAGE assembly with a k-mer of 31.

3.2 *Plasmodium falciparum*

The *P. falciparum de novo* assemblies were generated by Velvet version 1.2.03 to assemble 625bp insert Illumina reads (accession number ERR034295), using k-mers of 41, 45, 51, 55, 61, 65 and 71. The options `-ins_length 625 -exp_cov auto -cov_cutoff 10` were used when running `velvetg`. Each assembly was then subjected to three rounds of iterative scaffolding with SSPACE version 2 using the 625b insert, 3kb insert Illumina (ERR163027-9) and 454 8kb insert reads (ERR102953-4) in turn. The scaffolding was run using exactly the same method as that of *S. aureus* for each set of reads, iteratively decreasing the `-k` option. The 454 reads were mapped with SMALT version 0.6.2 with `SMALT index` options `-k 13 -s 2` and `SMALT map` options `-x -r 1 -i 15000 -y 0.8`.

During manual verification of the error calls made by REAPR, eight mis-assemblies were identified that were missed by REAPR (examples are shown in Figure S12), all of which were caused by repeats. Three errors were false joins caused by repeats of length 3.5kb, 7.5kb and 18kb. The error caused by the 3.5kb repeat is shown in Figure S12(a),(b) where it can be seen that the score is less than one (and so the corresponding bases are not error-free) at the breakpoint. The low score was over a stretch that was shorter than the window length used for error calling, therefore this error would be called using more aggressive parameters. The other two false joins could be called using a significantly larger fragment size library, for example 30kb fragments to call the join caused by the 18kb repeat. There were two short contigs

incorrectly inserted into the assembly, both of which were flagged when REAPR was run using the Illumina reads with an insert size of 625bp. The next two errors consisted of extremely repetitive scaffold ends, containing many gaps, corresponding to subtelomeric regions of the genome that are known to cause assembly problems. One example is shown in Figure S12c, where sufficiently long and overlapping stretches of correctly assembled sequence mean that no error is called. However, both regions contained gaps that were flagged as incorrect by the Illumina 625bp insert reads. The final error consisted of an inversion and collapsed repeat (Figure S12d), which would be possible to call using a library with more uniform read coverage.

In order to analyse versions 2.1.4 and 3 of the reference genome, REAPR was run twice. All read mapping was performed using SMALT, with `SMALT index` option `-k 13`. Both runs of REAPR used the Illumina reads to generate perfect and uniquely mapped read depth (with an insert size of 625 in the call to `reapr perfectmap`). The first run of REAPR used the Illumina reads to call collapsed repeats, and the second run used the 454 reads to call all other error types. The options `-s 2` and `-s 4` were used in `SMALT map` for the Illumina reads and 454 reads respectively.

The collapsed repeat calls in version 3 of the reference sequence are listed in Table S5. Although there is some overlap between the call sets, the Illumina data are more reliable due to the low coverage of the 454 data. Figure S4 shows details of all the confirmed collapsed repeats in version 3 of the genome. The collapsed repeat called in chromosome 9 at bases 974450 to 975508 was verified by PCR, were it was found that there should be two copies of the sequence. This region contains the GTP-CH gene, which has been previously reported to be present in three copies in the genome. Since the optical map only suggested that there should be one copy of this sequence, the evidence points to this region being highly variable between clones.

The PCR reaction to verify the collapsed repeat in chromosome 9 was performed using the NEB LongAmp Taq PCR kit. Reactions were cycled on a MJ Research Tetrad PTC 225 Thermal Cycler using a standard thermocycling consisting of an initial denaturation at 95°C for 30s, followed by 30 cycles each of 9°C for 10s, annealing at 5°C for 30s, and synthesis at 6°C for 6mins, and a final extension step at 6°C for 10mins. Products were electrophoresed and visualised using a FlashGel® System (Lonza).

REAPR only called five errors in version 3 of the *P. falciparum* reference genome, all of which were FCD errors. The region with the largest FCD error (0.75) was in chromosome 9 at position 1385281 to 1395288. Natural deletions are known to be commonplace in this region and this is confirmed by optical map (Figure S4e). The remaining error calls were likely to be false-positives, with FCD errors ranging from 0.54 to 0.6 just passing the FCD error cut-off of 0.49.

3.3 *Caenorhabditis elegans*

Illumina Lanes (ERR068453-6) were combined and then used to locate FCD errors within the *C. elegans* genome. Adapter sequences were searched for in these reads using SSAHA2 version 2.5.4, with the options `-best 1 -kmer 9 -skip 2 -seeds 2 -score 9 -cmatch 9 -ckmer 5`. The output was used to trim adapter sequences from the reads, discarding any read pairs with one or both mates having length less

than 35bp. The reference genome was indexed with `SMALT index -k 15 -s 4` and the options `-r 0 -x -i 20000 -y 0.5` were passed to `SMALT map`. Perfect uniquely mapping coverage across the genome was generated from a merge of three Illumina lanes (ERR103053-5) using the `perfectmap` function of REAPR with the insert size set to 265.

PCR primers were designed to amplify the top 20 FCD error regions using AcePrimer 1.3. PCR amplifications were performed with GoTaq Long PCR Master Mix (Promega) under the following conditions: initial denaturation at 94°C for 2 minutes, followed by 35 cycles of 95°C for 15 sec and 65°C for 12 min, and a final extension at 72°C for 10 min. The PCR products were visualised on a 1% agarose gel with TAE under UV light after staining with ethidium bromide. The PCR images are shown in Figure S6, the full details of these regions are in Table S6. For many of the 20 regions, more than one pair of primers was designed, because the regions were expected to be difficult to PCR. As remarked in the main text, the read and fragment coverage across the genome was extremely uneven. A typical region is shown in Figure S5.

3.4 *Mus musculus*

Short insert Illumina data from lanes SRR0676: 01, 05, 06, 11, 12, 15, 16, 20, 22–25, 33–36, 41, 45, 46, 48–50, 53, and 70 were mapped to the reference genome using SMALT. Options `-k 13 -s 2` were used when indexing the genome, and options `-r 1 -x -y 0.9 -i 500` were used when mapping. The resulting BAM file was used to generate perfect and uniquely mapped coverage across the genome using the `perfectfrombam` function of REAPR with the following command.

```
reapr perfectfrombam all.merged.bam 50 400 4 101 out_prefix
```

Large insert Illumina data from lanes SRR0676: 03, 04, 07, 10, 18, 19, 31, 39, 44, 52, 54, 57, 58, 60, 63 and 69 were also mapped to the reference genome using SMALT. The genome was indexed using `-k 13 -s 2` and reads were mapped with options `-r 1 -x -y 0.5 -i 5000`.

3.5 *Homo sapiens*

Short insert Illumina data from lanes SRR067577–9 and SRR0677: 80, 84, 85, 87, 89 and 91–93 were mapped to the reference genome using SMALT, using identical settings to those used when mapping the *M. musculus* short insert data. The resulting BAM file was used to generate perfect and uniquely mapped coverage across the genome using the `perfectfrombam` function of REAPR with the following command.

```
reapr perfectfrombam all.merged.bam 50 300 4 101 out_prefix
```

Large insert Illumina data from lanes SRR0677: 71, 73, 76, 77, 78, 79, 81 and 86 were mapped to the reference genome using the same settings as those used when mapping the *M. musculus* large insert reads.

3.6 *Brugia pahangi*

We used REAPR to analyse the progress of the ongoing genome project of the helminth *Brugia pahangi* (Figure 3). The assembly was made from two lanes of Illumina paired reads – one short insert library (ERR070030) and a 3kbp insert size library (ERR068352). The overall contiguity improvement is clear when measuring the contiguity with the N50 after improvement by REAPR, in contrast to the original N50 of each assembly. The four stages at which the assembly was analyzed were as follows.

1. An initial assembly using Velvet.

2. Scaffolding using SSPACE.

3. Gap filling using IMAGE. The resulting scaffolds were split into contigs by breaking at every gap, then used as input to SSPACE for a second round of scaffolding.

4. Gap filling using the tool GapFiller.

4 Figures and tables

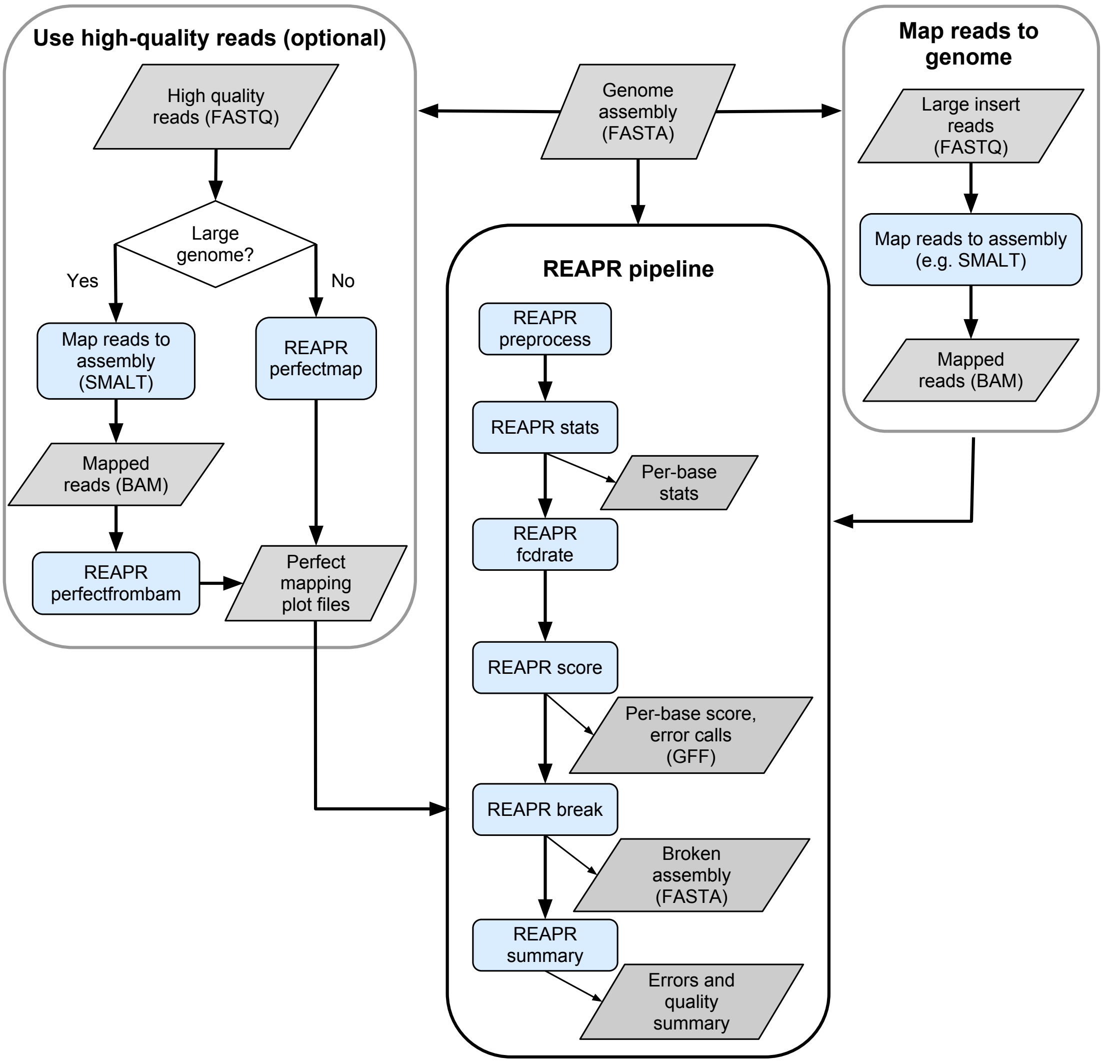

Figure S1: REAPR pipeline. Boxes with a blue background denote REAPR functions. The 'pipeline' function is a shortcut for ease of use for the end user, which runs the functions `preprocess`, `stats`, `fcdrate`, `score`, `break` and `summary` in sequence using the default settings.

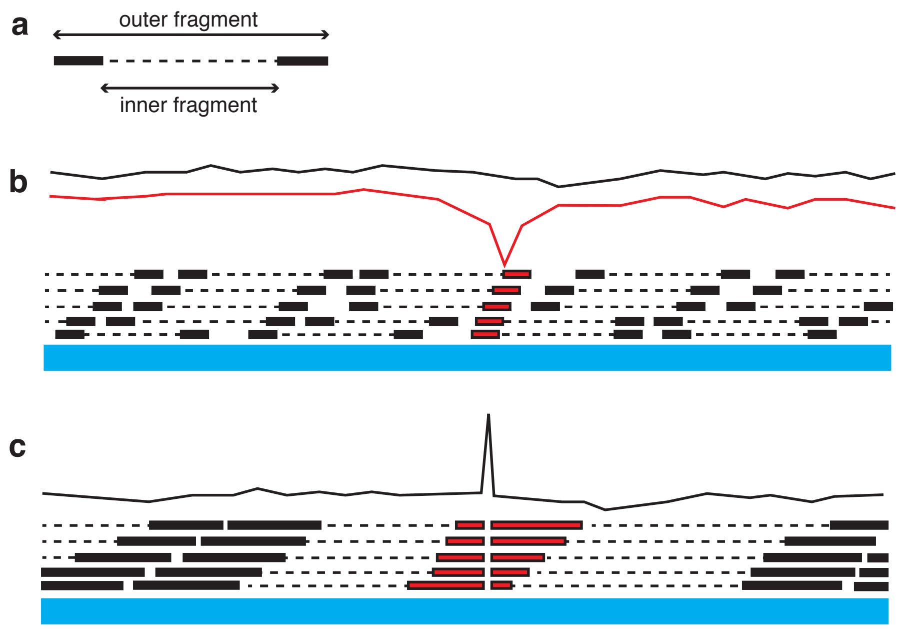

Figure S2: Inner and outer fragments, and soft-clipping. (a) The difference between an inner and outer fragment for a read pair. Two reads are shown (black rectangles), joined by a dashed line to indicate that they comprise a read pair. Outer fragment includes the reads, whereas inner fragment does not. (b) The difference between inner (shown in red) and outer fragment coverage (shown in black) plots, over a breakpoint. The reads shown in red span the breakpoint, which frequently occurs over a poly(A) tract or other repetitive sequence. The outer fragment coverage does not significantly drop, but the inner fragment coverage drops to zero since this plot does not include the reads themselves. (c) An example of a soft-clipping plot. The red reads have been soft-clipped in order to align to the genome. A small deletion in the genome is a common cause of a stack of soft-clipped reads.

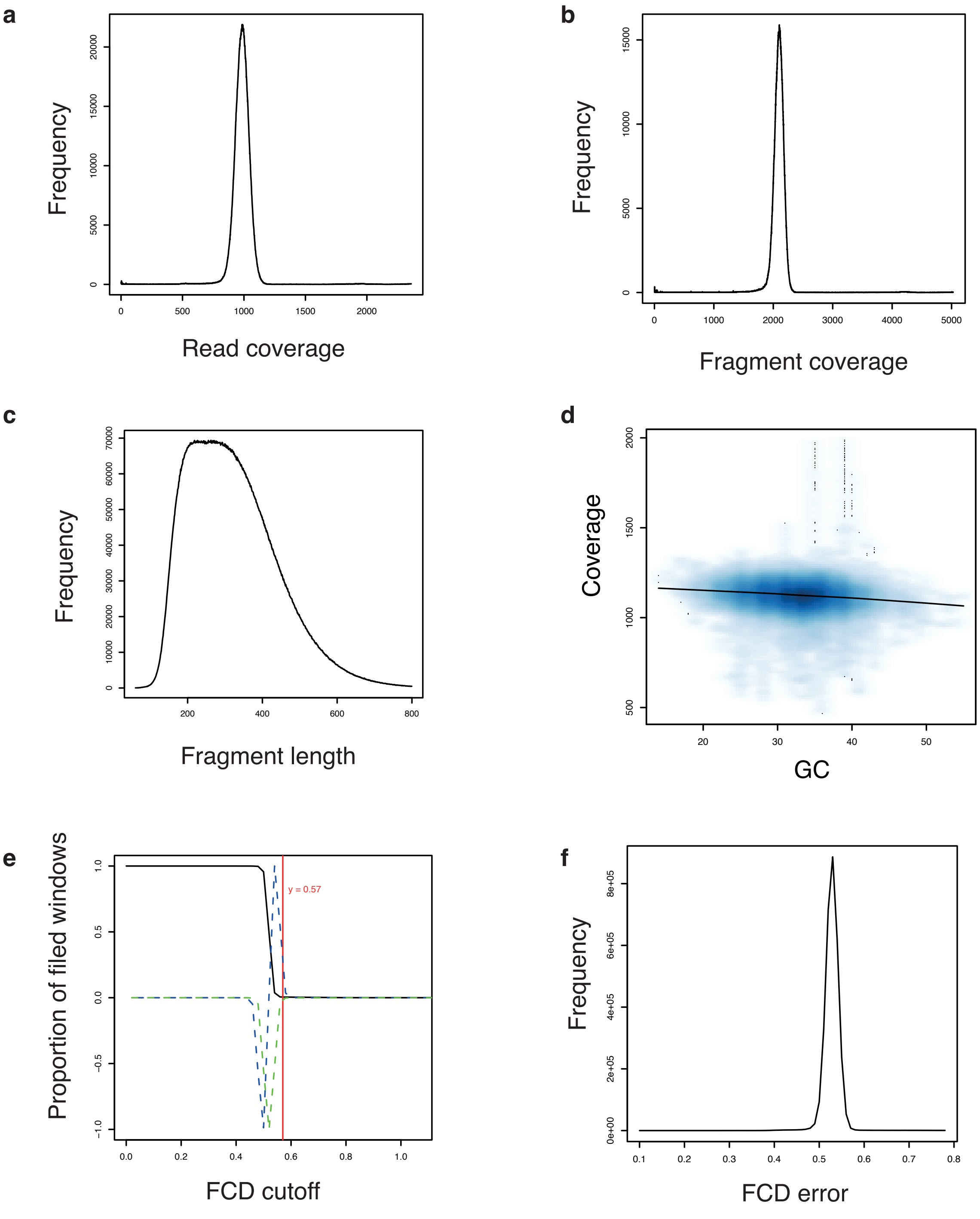

Figure S3: Plots generated by REAPR after running on the *S. aureus* Velvet assembly. The read (a) and fragment (b) per-base coverage distribution of the assembly. (c) The fragment length distribution, determined by mapped read pairs. (d) LOWESS line through a scatter plot of fragment coverage versus GC content. (e) Calculation of the FCD error cutoff. (f) Distribution of per-base FCD error.

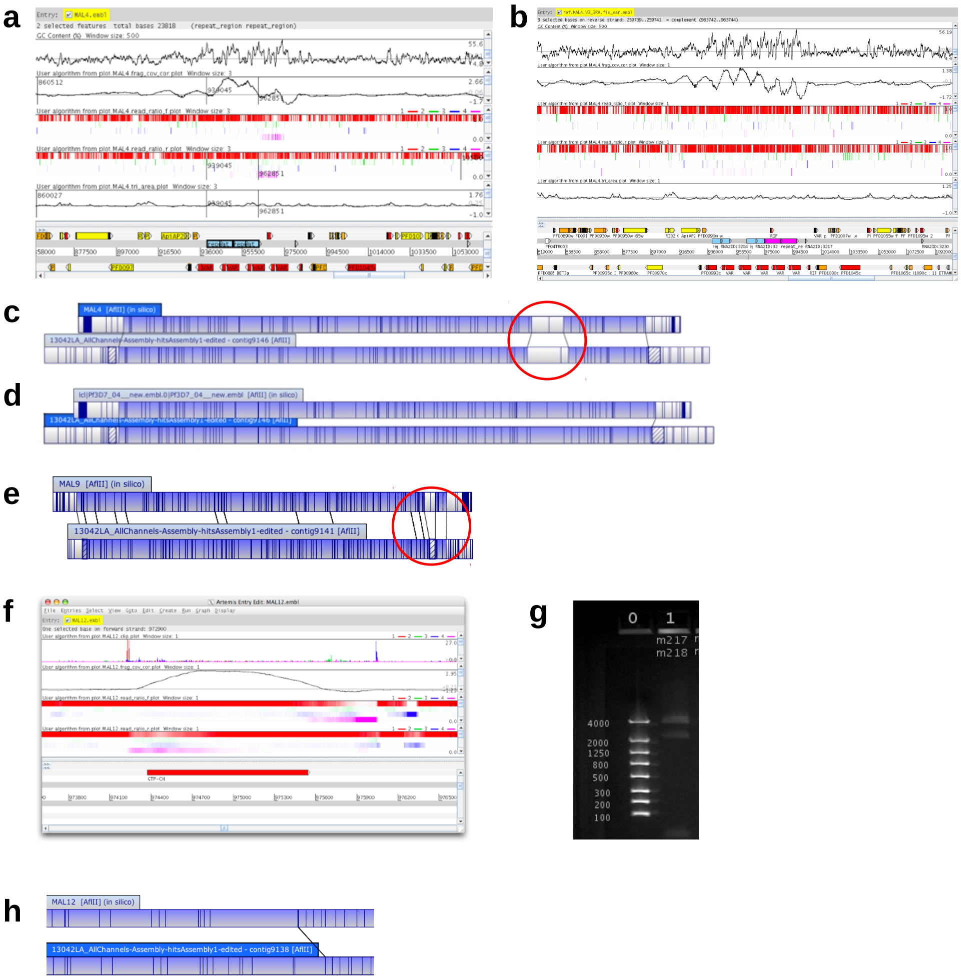

Figure S4: REAPR error calls and confirmation in the *Plasmodium falciparum* assembly v3. (a) Collapsed repeat (blue rectangles) in chromosome 4. The top plot shows the GC content, below is the relative error in inner fragment coverage. The heatmaps show the type of read coverage on each strand: proper read pairs (red), orphaned reads (green), wrong insert size (blue) and wrong orientation (purple). The bottom plot shows the FCD error. (b) As (A), but with the sequence fixed by expanding the repeat. (c) Optical map (bottom) aligned to an *in silico* digest of chromosome 4 (top). The area marked with a red circle is the region shown in (a). (d) As (c), but with the corrected sequence. (e) Optical map (bottom) aligned to *in silico* digest (top) of chromosome 9 with a red circle marking location 1385281 to 1395288, called as an FCD error. (f) Chromosome 12, showing the repeat at position 974450-975508. The top plot is the number of soft-clipped reads. The second plot shows the relative error in fragment coverage. The heatmaps follow the same scheme as in (a). (g) PCR of (f) confirming the collapsed repeat by showing two bands: one at the expected length and a second at twice the expected length. (H) Optical map confirming the collapsed repeat called in chromosome 12 at approximate position 1,710,000 (layout identical to (C)).

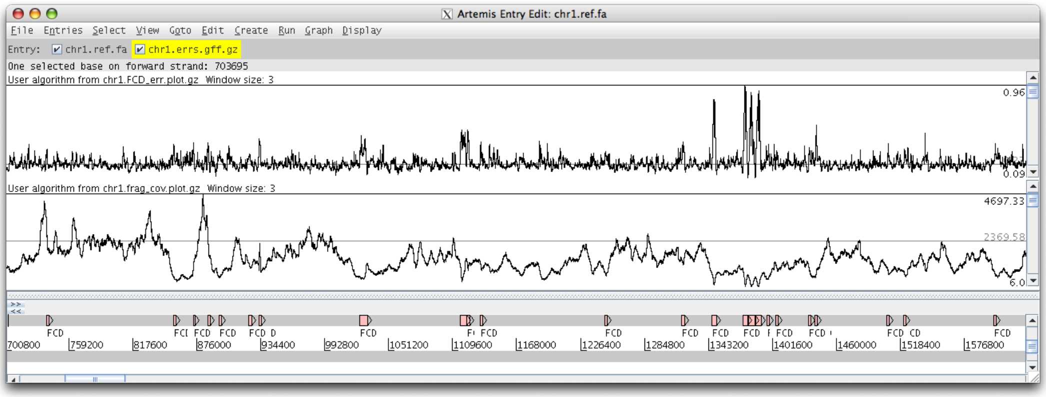

Figure S5: A region of the *C. elegans* genome showing uneven coverage and resulting FCD error calls. The top plot is the FCD error and lower plot is the fragment coverage, which varies between zero and 4,700. FCD error calls are marked with pink boxes. The region at approximately 138,000 was confirmed as an error by PCR.

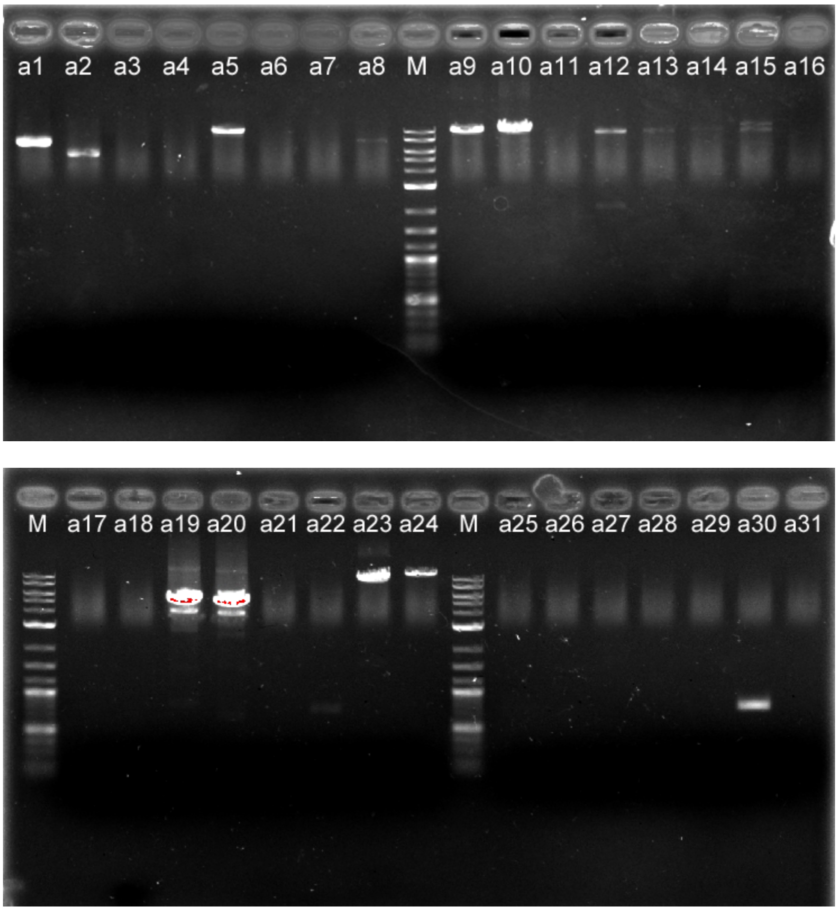

Figure S6: PCR images of top 20 FCD errors identified in the *C. elegans* reference genome. Full details can be found in Table S6.

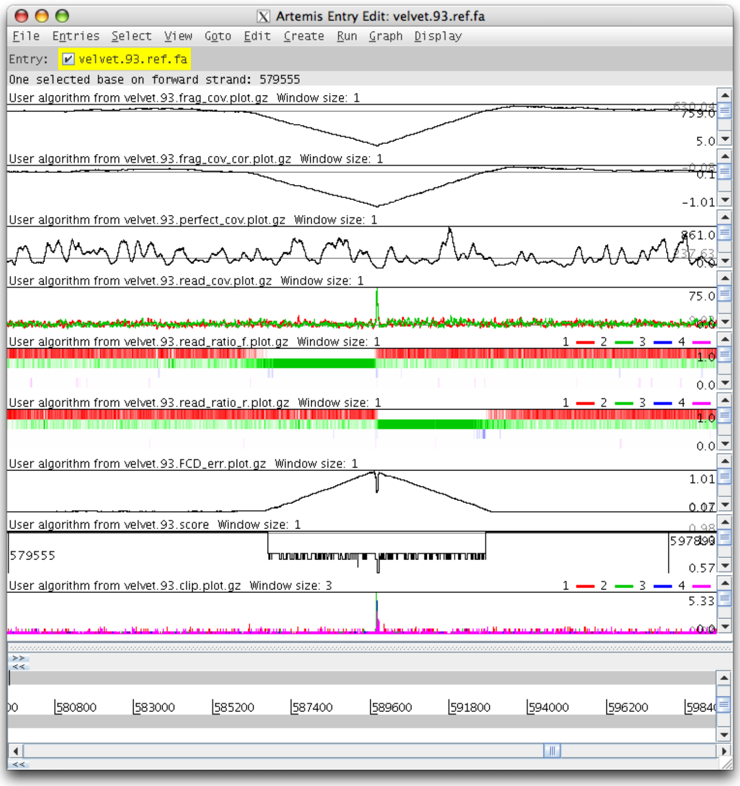

a Fragment coverage

b Fragment coverage error

c Perfect unique read coverage

d Read coverage

e Read type, forward strand

f Read type, reverse strand

g FCD error

h Score

i Soft clipping

Figure S7: Artemis screenshot showing all the plots made by REAPR, in the region where a contig error was called due to an FCD error. The read coverage plot (d) shows the forwards strand in red and the reverse strand in green. (e) and (f) show the type of read coverage on the forwards and reverse strands respectively (same color scheme as that of Figure S4). The soft clipping pileup in (i) is broken down into the four combinations of reads clipped at their left or right ends and on each strand.

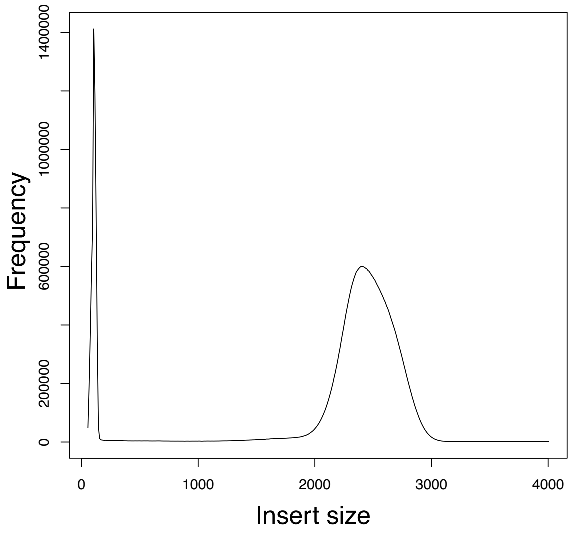

Figure S8: Insert size plot of SRR067771 determined by mapping to the human reference genome.

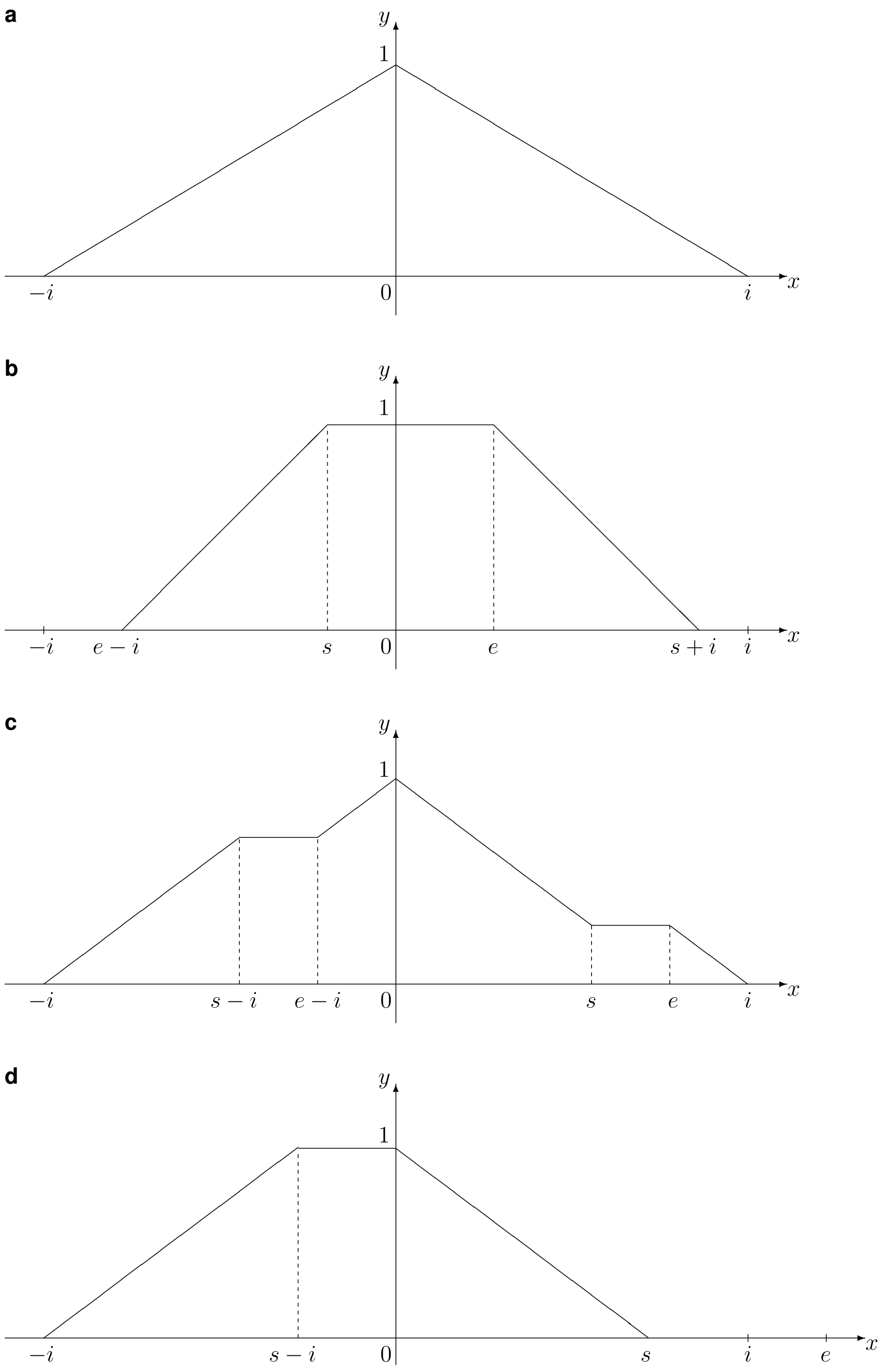

Figure S9: Ideal FCD plots, depending on where the gap (if any) lies in relation to the centre of the plot. The notation used is i=insert size, s=start position of gap, e=end position of gap. (a) No gap. (b) A gap over the centre of the plot. (c) A gap less than one insert size away from the centre (but not on the centre). (d) A gap within an insert size of the centre, but extending away from the centre.

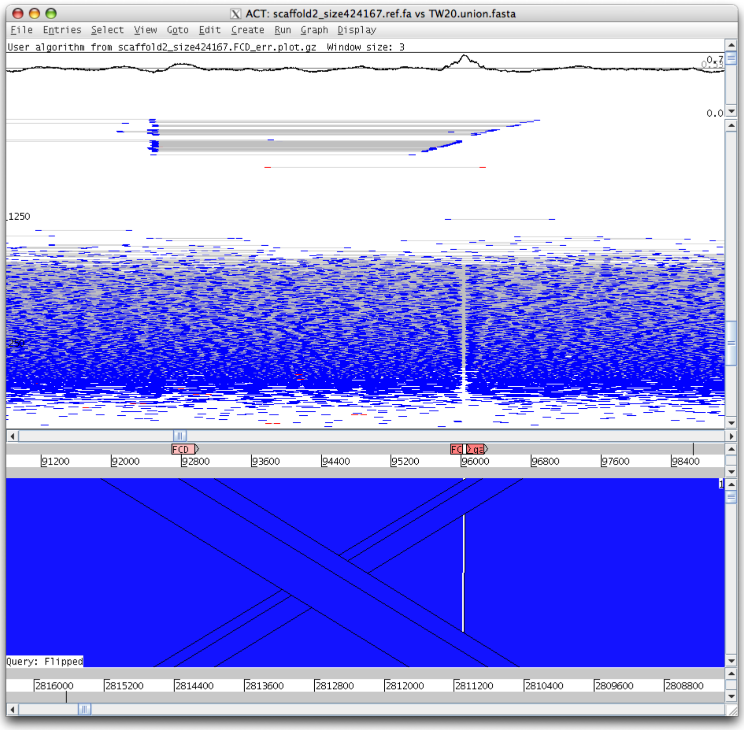

Figure S10: ACT screenshot of two typical false-positive REAPR errors calls, in the *S. aureus de novo* assembly. The bottom genome is the reference, the upper genome is Scaffold2 of the assembly, with blast hits shown in blue between the two sequences. Read pairs are shown in blue, joined by grey lines, with their height in the figure determined by their insert size. Note the set of read pairs mapped to the repetitive sequences with a higher than expected insert size. These caused two FCD errors, shown in pink on the assembly scaffold (the error on the right hand side contains a gap marked in white) with the corresponding peaks visible in the FCD error plot at the top of the figure.

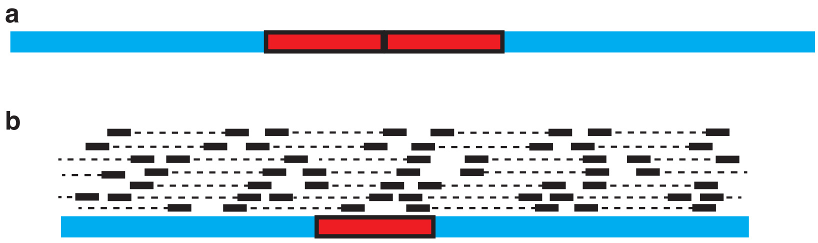

Figure S11: Schematic of how perfect and unique coverage can remain non-zero over a collapsed repeat. (a) The correct sequence, with two copies of a tandem repeat shown in red. (b) The assembly with the repeat sequence collapsed. Read pairs can still map so that the assembly has perfect and unique coverage across the entire region, despite no fragment spanning across the collapsed repeat (i.e. one read mapped to the left of the repeat and its mate mapped to the right of the repeat).

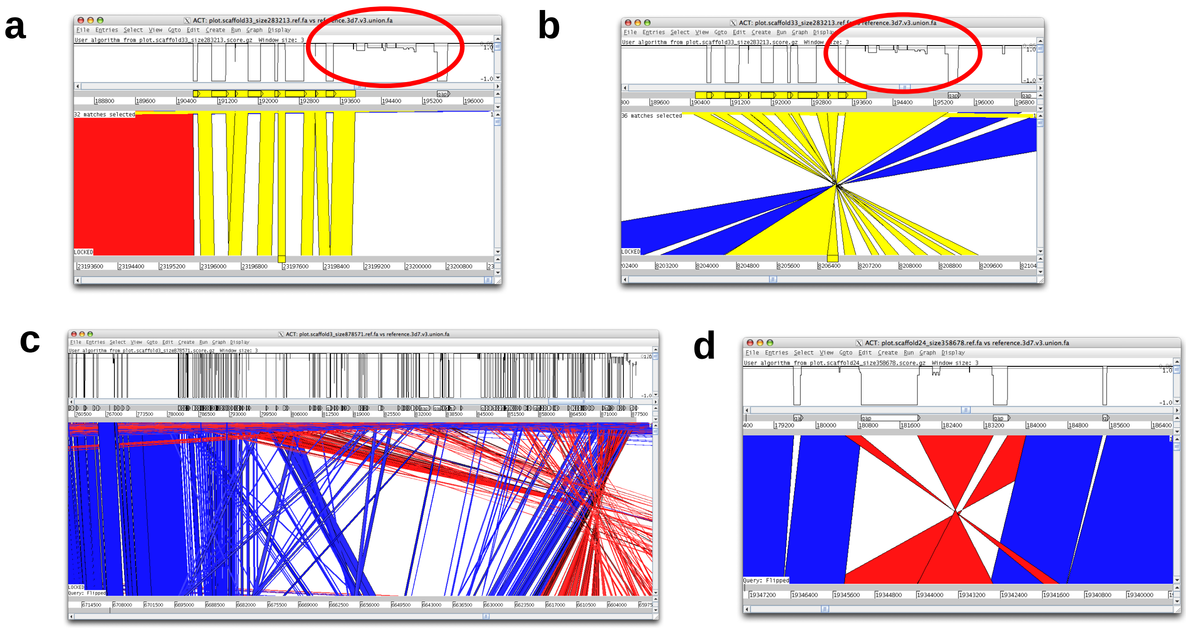

Figure S12: Example false-negative errors in the *P. falciparum de novo* assembly with k-mer 55. In all images, the top genome is the assembly and the bottom is the reference sequence, with BLAST hits shown between the two. The plot shown is the REAPR score across the assembly (where gaps are given a score of -1) with the low scoring bases circled in red. (a),(b) A false join, with the same region of the assembly shown in (a) and (b) but matching to different regions of the reference. There is 3.5kb of sequence in common (shown in yellow) that caused the mis-assembly. (c) A messy scaffold end, with mis-assemblies caused by repeats. (d) An inversion and collapsed repeat.

Organism	Assembly	GC(%)	Length	Gaps Number	Gaps Length	Perfect unique reads Fragment length	Perfect unique reads Read coverage	Structural errors reads Fragment length	Structural errors reads Read coverage	Structural errors reads Fragment coverage	N50	Corrected N50
S. aureus	GAGE Reference	32	2,903,081	0	0	165	145	3,400	14	672	2,872,915	2,872,915
S. aureus	Allpaths	32	2,880,676	48	9,900	165	145	3,400	14	661	1,091,731	689,008
S. aureus	Bambus2	32	2,862,930	99	29,205	165	145	3,400	14	676	1,083,792	248,173
S. aureus	MSR-CA	33	2,872,905	81	10,353	165	145	3,400	14	663	2,411,914	605,467
S. aureus	SOAPdenovo	32	2,924,135	9	4,857	165	145	3,400	14	683	331,598	288,184
S. aureus	ABySS	39	3,893,185	69	55,885	165	141	3,400	8	668	26,537	25,368
S. aureus	Velvet	31	2,877,995	128	17,688	165	145	3,400	14	667	762,333	212,605
S. aureus	SGA	30	3,128,388	654	300,607	165	145	3,400	13	662	149,421	19,484
S. aureus	TW20 de novo $k31$	34	3,014,840	104	2,644	265	651	265	990	2,102	158,791	94,293
S. aureus	TW20 de novo $k41$	34	3,017,555	62	2,328	265	651	265	990	2,102	171,786	138,690
S. aureus	TW20 de novo $k51$	33	3,017,412	55	2,260	265	651	265	990	2,102	206,359	120,333
S. aureus	TW20 de novo $k61$	33	3,020,286	61	1,143	265	651	265	990	2,102	172,013	140,944
S. aureus	TW20 de novo $k71$	33	3,021,434	31	249	265	651	265	985	2,102	206,425	172,033
S. aureus	TW20 reference	32	3,075,806	0	0	265	651	265	985	2,102	3,043,210	3,043,210
P. falciparum	*de novo k41*	20	25,012,772	18,363	3,379,558	625	67	7,745	1	49	54,936	33,590
P. falciparum	*de novo k45*	21	24,492,830	14,651	2,905,493	625	74	7,745	1	73	92,222	53,307
P. falciparum	*de novo k51*	21	24,110,585	11,810	2,679,903	625	74	7,745	1	80	148,387	99,204
P. falciparum	*de novo k55*	22	23,815,661	11,636	2,638,349	625	74	7,745	1	81	422,542	250,768
P. falciparum	*de novo k61*	21	23,742,618	10,791	3,366,469	625	75	7,745	1	72	184,532	163,141
P. falciparum	*de novo k65*	21	23,536,180	10,255	4,412,828	625	76	7,745	1	70	109,329	108,841
P. falciparum	*de novo k71*	21	19,363,824	7,637	6,233,940	625	77	7,745	1	29	31,606	31,568
P. falciparum	Version 2.1.4	19	23,299,734	160	947	625	74	7,745	1	101	1,687,655	1,687,655
P. falciparum	Version 3	19	23,328,019	0	0	625	74	7,745	1	108	1,687,656	1,687,656
C. elegans	WS228	35	100,286,070	0	0	265	19	2,445	166	2664	17,493,793	17,493,793
M. musculus	GRCm38	39	2,725,521,370	522	77,999,939	185	11	2,045	18	245	130,694,993	100,237,891
H. sapiens	GRCh37	41	3,095,677,412	360	234,350,278	165	19	2,885	17	265	155,270,560	146,364,022

Table S1: Summary of all genomes analysed by REAPR. The corrected N50 is the N50 of scaffolds after breaking the assembly at scaffolding errors identified by REAPR.

Organism	Assembly	Contig Errors				Scaffold Errors				REAPR base type count		
		FCD	Low fragment coverage	False positives	False negatives	FCD	Low fragment coverage	False positives	False negatives	Error free	Repetitive	Error free and repetitive
S. aureus	GAGE Reference	2	0	2	ND	NA	NA	NA	NA	2,837,518	66,980	41,475
S. aureus	Allpaths	0	0	ND	ND	2	0	ND	ND	2,793,380	55,175	34,551
S. aureus	Bambus2	4	0	ND	ND	13	0	ND	ND	2,685,529	29,926	29,234
S. aureus	MSR-CA	7	0	ND	ND	7	1	ND	ND	2,715,538	75,783	36,513
S. aureus	SOAPdenovo	16	0	ND	ND	1	0	ND	ND	2,601,080	131,001	50,902
S. aureus	ABySS	10	0	ND	ND	24	0	ND	ND	1,554,076	1,419,367	65,823
S. aureus	Velvet	8	1	1	18	11	13	0	1	2,574,955	49,188	35,178
S. aureus	SGA	10	0	ND	ND	153	0	ND	ND	2,095,459	127,994	123,566
S. aureus	TW20 de novo $k31$	6	0	ND	ND	35	0	ND	ND	2,936,299	112,193	97,619
S. aureus	TW20 de novo $k41$	6	0	ND	ND	15	0	ND	ND	2,952,491	70,812	61,365
S. aureus	TW20 de novo $k51$	5	0	ND	ND	17	0	ND	ND	2,958,285	56,186	50,407
S. aureus	TW20 de novo $k61$	2	0	ND	ND	30	0	ND	ND	2,961,464	51,074	47,344
S. aureus	TW20 de novo $k71$	6	0	6	1	18	0	2	0	2,965,746	49,005	45,750
S. aureus	TW20 reference	6	0	6	0	NA	NA	NA	NA	3,010,572	126,916	71,158
P. falciparum	*de novo k41*	4	4	ND	ND	0	237	ND	ND	19,006,184	7,278,941	6,258,958
P. falciparum	*de novo k45*	6	5	ND	ND	30	162	ND	ND	17,284,536	6,101,786	5,393,596
P. falciparum	*de novo k51*	4	4	ND	ND	46	58	ND	ND	18,021,775	5,215,876	4,740,439
P. falciparum	*de novo k55*	6	6	0	ND	24	32	1	8	19,340,437	4,801,419	4,432,088
P. falciparum	*de novo k61*	3	3	ND	ND	8	3	ND	ND	18,192,651	4,720,028	4,369,896
P. falciparum	*de novo k65*	3	3	ND	ND	5	2	ND	ND	16,872,331	4,459,636	4,142,873
P. falciparum	*de novo k71*	0	0	ND	ND	1	1	ND	ND	10,821,723	3,598,515	3,350,249
P. falciparum	Version 2.1.4	4	0	3	0	1	3	1	0	22,023,362	2,553,079	2,233,601
P. falciparum	Version 3	5	0	4	0	NA	NA	NA	0	22,128,358	2,531,685	2,246,154
C. elegans	WS228	848	6	ND	ND	NA	NA	NA	NA	90,580,995	6,669,748	3,632,983
M. musculus	GRCm38	22,611	1,160	ND	ND	31	10	ND	ND	2,183,123,682	268,171,234	102,815,661
H. sapiens	GRCh37	6,034	625	ND	ND	6	0	ND	ND	2,447,229,618	206,305,069	107,060,403

Table S2: Error calls made by REAPR. Scaffold errors are not applicable where an assembly conatins no gaps.

Organism	Assembly	Link	Low score	Soft clip	Low read coverage	Low perfect read coverage	Repeat	Improper read pairs
S. aureus	GAGE Reference	8	0	0	0	38	0	42
S. aureus	Allpaths	43	0	4	0	32	0	48
S. aureus	Bambus2	82	0	77	0	33	0	112
S. aureus	MSR-CA	68	5	3	0	48	0	146
S. aureus	SOAPdenovo	225	3	34	0	198	0	93
S. aureus	ABySS	379	188	8	6	3,804	0	752
S. aureus	Velvet	291	0	2	0	153	0	148
S. aureus	SGA	1,302	0	6	0	19	0	70
S. aureus	TW20 de novo $k31$	105	0	1	0	190	14	17
S. aureus	TW20 de novo $k41$	105	0	0	0	85	18	10
S. aureus	TW20 de novo $k51$	115	0	0	0	34	17	17
S. aureus	TW20 de novo $k61$	85	0	0	0	20	18	7
S. aureus	TW20 de novo $k71$	85	0	0	0	16	16	3
S. aureus	TW20 reference	0	0	0	0	44	2	0
P. falciparum	*de novo k41*	20,903	356	170	8,460	8,139	73	1,761
P. falciparum	*de novo k45*	15,981	912	151	9,333	6,150	34	1,555
P. falciparum	*de novo k51*	10,238	628	120	9,816	3,933	13	1,704
P. falciparum	*de novo k55*	4,888	295	118	9,179	2,475	30	2,660
P. falciparum	*de novo k61*	9,288	344	107	8,370	2,549	64	2,566
P. falciparum	*de novo k65*	11,561	262	98	6,790	2285	18	2,889
P. falciparum	*de novo k71*	14,119	62	92	2,732	1,499	112	2,551
P. falciparum	Version 2.1.4	794	120	58	17,057	5,294	17	731
P. falciparum	Version 3	713	97	57	17,110	4,923	19	655
C. elegans	WS228	348	4	132	23	34,297	43	1139
M. musculus	GRCm38	38,830	34,158	23,355	52,016	1,669,216	898	1,146,275
H. sapiens	GRCh37	64,529	18,897	37,038	18,028	2,212,778	341	2,357,906

Table S3: Warnings reported by REAPR.

Version	Chromosome	Reason	Error type	Start	End	Mean FCD error
2.1.4	MAL7	No fragment coverage	Scaffold	116,360	117,879	NA
2.1.4	MAL8	No fragment coverage	Scaffold	1,347,480	1,347,843	NA
2.1.4	MAL9	FCD error	Contig	1,385,281	1,395,256	0.74
2.1.4	MAL11	FCD error	Scaffold	315,762	320,457	0.58
2.1.4	MAL11	FCD error	Contig	1,939,993	1,945,040	0.54
2.1.4	MAL12	FCD error	Contig	230,065	236,936	0.59
2.1.4	MAL13	No fragment coverage	Scaffold	2,805,738	2,806,296	NA
2.1.4	MAL14	FCD error	Contig	313,825	318,248	0.56
3	MAL9	FCD error	Contig	1,385,281	1,395,288	0.75
3	MAL11	FCD error	Contig	315,737	320,480	0.59
3	MAL11	FCD error	Contig	1,939,681	1,945,072	0.54
3	MAL12	FCD error	Contig	229,985	236,984	0.60
3	MAL14	FCD error	Contig	313,801	318,272	0.57

Table S4: Error calls made by REAPR in versions 2.1.4 and 3 of the reference genome of *Plasmodium falciparum*.

Calls using Illumina data

Chromosome	Start	End	Coverage
4	547,967	548,078	2.1
4	939,862	940,654	2.3
4	943,362	943,854	2.4
4	945,630	946,397	3.0
4	947,074	947,335	2.0
4	947,533	948,327	2.4
4	949,060	949,977	2.7
4	951,777	952,555	2.3
4	955,224	955,791	2.4
4	957,524	958,339	2.9
4	959,095	959,335	2.1
4	959,360	960,234	2.5
4	960,972	961,875	2.8
6	16,747	16,862	2.1
9	63,764	63,988	2.1
10	1,617,320	1,617,856	2.2
10	1,618,178	1,618,853	2.7
10	1,621,926	1,622,192	2.1
10	1,638,603	1,638,812	2.1
11	65,520	65,819	2.3
11	1,922,836	1,923,239	2.4
12	974,450	975,508	4.5
12	1,708,884	1,709,055	2.0
12	1,710,511	1,711,133	2.4
12	1,712,313	1,712,556	2.2
12	1,713,028	1,713,232	2.2
12	1,714,977	1,715,493	2.3
13	2,378,926	2,379,198	2.1
13	2,796,893	2,797,277	2.4

Calls using 454 data

Chromosome	Start	End	Coverage
1	9,385	21,838	3.3
1	625,970	628,970	2.3
2	930,747	931,958	2.1
3	16,535	16,848	2.0
3	16,913	20,020	2.2
3	20,040	23,339	2.1
3	23,370	24,429	2.0
3	24,440	26,024	2.0
4	946,041	952,353	2.4
5	15,052	15,198	2.0
5	15,352	15,582	2.0
5	15,728	15,840	2.0
10	15,423	18,208	2.2
10	1,661,576	1,661,682	2.0
10	1,662,031	1,663,430	2.1
10	1,664,385	1,665,139	2.1
10	1,665,335	1,674,551	2.3
13	15,073	15,580	2.1
13	15,593	15,710	2.0

Table S5: Collapsed repeat calls in *P. falciparum* version 3, excluding those in the mitochondria and apicoplast. The coverage columns show the mean fragment coverage in the region as a multiple of the expected coverage.

Chr	FCD error			Primers					PCR size		Wormbase annotation feature count					
	Start	End	Mean error	ID	Position 1 (k)	Position 2 (k)	Sequence 1	Sequence 2	Expected	Actual (k)	Gene	Tandem repeat	Inverted repeat	Repeat region	TE	TE insertion site
IV	2,372,755	2,377,056	0.64	a1	2,371.5	2,378.9	gtcacaacttggccaaacct	tcacttttccacacagctcg	7,436	7.5	1	2	13	11	0	1
IV	2,372,755	2,377,056	0.64	a2	2,372.6	2,378.5	acaaatttcgattttaagggtgaa	ttttcatcacaatttgctcttgtt	5,970	6.1	1	2	13	11	0	1
X	214,495	218,796	0.66	a3	212.1	219.5	agccattggtggtaacgaag	atgacaaaggaagggcacac	7,476	failed	2	8	2	7	0	3
I	134,6539	1,349,694	0.66	a4	1,346.4	1,350.1	ggaagaatgtgggggaaaat	gttttacagcggggttcaaa	3,716	failed	1	2	257	6	0	0
I	138,6223	1,390,464	0.68	a5	1,387.7	1,390.0	agtcaacttccaaagggctg	atgccaaaacgctaaattcg	2,291	8.8	1	0	5	11	0	0
I	138,6223	1,390,464	0.68	a6	1,386.2	1,391.1	cagccgattcgaaacaattt	cgcggaatatcgagaagaaa	4,941	failed	1	0	5	11	0	0
I	138,6223	1,390,464	0.68	a7	1,385.8	1,390.6	ccaaaaatgctgaacttttagtcc	cggaaaaatcgaagaaaactagaa	4,967	failed	1	0	5	11	0	0
X	1,1309,167	11,312,598	0.68	a8	11,310.1	11,311.6	cctttggaataaacctgcga	cctggtttcaaccttgtcgt	1,534	7.5	1	0	1	1	2	0
X	1,1309,167	11,312,598	0.68	a9	11,308.8	11,312.6	tccaaacattgtcgacagga	tttgcggaccaaacattaca	3,792	8.8	1	0	1	1	2	0
X	1,1309,167	11,312,598	0.68	a10	11,308.9	11,312.8	actatctgcttttcgttccaaatc	gctatacaaaggtcttcagccaat	3,900	9.1	1	0	1	1	2	0
IV	15,074,113	15,082,149	0.69	a11	15,073.8	15,082.6	cctcagacctcatggcattt	tttttctcactgttggcgtg	8,960	failed	4	21	15	13	0	0
V	6,093,706	6,096,933	0.69								1	3	5	4	0	0
IV	1,287,271	1,290,690	0.69	a12	1,287.0	1,291.0	atcttttcgggcagttttcc	gaatttgagcgatttccagc	3,972	8.3,2.3	1	2	5	1	0	0
IV	1,287,271	1,290,690	0.69	a13	1,287.1	1,291.1	taagacagaagctcacatttctgg	ttcaatttttagcgattttcaaca	3,908	8.8	1	2	5	1	0	0
IV	1,287,271	1,290,690	0.69	a14	1,286.7	1,291.0	tttaaggaattgtcctctgtgtga	tcaatttttagcgattttcaacaa	4,303	8.9	1	2	5	1	0	0
II	1,3736,815	13,740,201	0.69	a15	13,736.7	13,740.3	cgactcgatttctggggtaa	aacagcaaaatgccgaaatc	3,614	10.1,8.8	1	7	4	4	0	1
II	1,3736,815	13,740,201	0.69	a16	13,736.7	13,740.6	tcgatttctggggtaatttattgt	tattaccgaaaaccaaaatatccg	3,919	failed	1	7	4	4	0	1
X	12,811	17,547	0.70	a17	12.7	18.5	gtgcgtatcatctcgagcaa	aaaaacaaacccacaaagcg	5,906	failed	1	4	9	11	0	0
X	12,811	17,547	0.70	a18	12.7	17.6	agcatcataatatcaacagtgcgt	gatttttcaagagaaaattccgat	4,965	failed	1	4	9	11	0	0
X	5,051,623	5,054,766	0.70								0	4	13	2	0	1
V	15,905,278	15,911,391	0.70	a19	15,905.0	15,911.9	tcggcagttagaaagtggct	cgcttgtgaactccaagtga	6,956	5.5,4.4	5	0	5	4	2	0
V	15,905,278	15,911,391	0.70	a20	15,905.4	15,912.1	attgaatgttcactgacgtgatct	taggaagaaggtcatcattgttca	6,780	5.3,4.3	5	0	5	4	2	0
IV	3,134,443	3,137,634	0.71	a21	3,134.0	3,137.7	tcggtacgagatactgtgcg	aatccacctcgtccactgac	3,721	failed	2	1	2	9	0	1
IV	3,134,443	3,137,634	0.71	a22	3,134.4	3,138.1	aaagaaacagttttggagcaattc	actacctaatgcctacctacacgc	3,775	0.7	2	1	2	9	0	1
I	8,453,143	8,456,850	0.72	a23	8,453.9	8,455.5	ttgcaagaatgatgaggctg	acgggatgtagcggttgtag	1,610	8.8	0	2	0	3	0	1
I	8,453,143	8,456,850	0.72	a24	8,452.9	8,456.9	aaaaggaagactcaacgaattttg	gcagtcaaagtagatggaaggaat	3,953	9.5	0	2	0	3	0	1
X	254,077	257,922	0.72	a25	254.9	256.9	tttttccgaactttgaacgg	cgagaaaaatagcgtttggc	2,016	failed	1	5	12	5	0	0
X	254,077	257,922	0.72	a26	253.7	258.3	ggtcgaaccaagacaatgct	gcaacaggtacaccggatct	4,643	failed	1	5	12	5	0	0
I	1,374,733	1,378,218	0.73	a27	1,375.7	1,377.9	ttcggtagcctcagctcaat	ttgttgtcgtcggaactcag	2,193	failed	2	4	57	9	0	1
I	1,374,733	1,378,218	0.73	a28	1,374.0	1,378.6	gggcaaaattagtttttaatgacg	ttagacttaggtttaggcattggc	4,685	failed	2	4	57	9	0	1
IV	3,168,076	3,171,903	0.73	a29	3,167.9	3,172.0	cggaaacactgtgccttttt	tcgaaggttctggaaccaag	3,168	failed	2	1	2	9	0	1
X	5,047,234	5,050,068	0.73								0	4	13	2	0	1
I	15,060,373	15,063,243	0.74								4	0	0	1	0	33
X	5,905,927	5,907,756	0.76	a30	5,905.9	5,908.1	aaaatgtacgacacgctccc	gtggtgacaatgagtccgtg	2,801	0.7	0	4	2	4	0	0
X	5,905,927	5,907,756	0.76	a31	5,905.3	5,908.9	acatccggatacgtttattaggaa	cgattagcagcatgatatcaaaac	3,560	failed	0	4	2	4	0	0

Table S6: Details of the *C. elegans* PCRs. Rows with empty data are regions where it was not possible to design PCR primers.

Organism	Assembly	Short insert SMALT		Short insert `perfectmap`		Large insert SMALT			REAPR	
		Mem (MB)	CPU (s)	Mem (MB)	CPU (s)	Mem (MB)	CPU (s)	BAM size (MB)	Mem (MB)	CPU (s)
S. aureus	GAGE Reference	NA	NA	232	202	642	1,014	163	68	279
S. aureus	Allpaths	NA	NA	221	366	681	1,016	163	43	275
S. aureus	Bambus2	NA	NA	219	358	668	1,018	163	43	270
S. aureus	MSR-CA	NA	NA	220	346	670	1,017	163	204	291
S. aureus	SOAPdenovo	NA	NA	224	344	1,069	1,023	163	57	269
S. aureus	ABySS	NA	NA	279	338	1,652	1,098	168	204	331
S. aureus	Velvet	NA	NA	223	376	1,018	1,025	163	34	287
S. aureus	SGA	NA	NA	223	356	641	1,014	163	24	302
S. aureus	TW20 de novo k31	NA	NA	236	375	10,438	1,041	2,974	29	484
S. aureus	TW20 de novo k41	NA	NA	224	369	8,187	1,027	2,973	157	469
S. aureus	TW20 de novo k51	NA	NA	230	353	7,429	1,065	2,974	203	532
S. aureus	TW20 de novo k61	NA	NA	240	363	7,091	1,003	2,974	30	484
S. aureus	TW20 de novo k71	NA	NA	231	374	6,996	1,631	2,974	29	482
S. aureus	TW20 reference	NA	NA	236	688	8,861	2,386	2,977	203	512
P. falciparum	de novo k41	NA	NA	1,385	952	1,270	142	99	159	1,942
P. falciparum	de novo k45	NA	NA	1,382	1,015	971	146	98	204	2,060
P. falciparum	de novo k51	NA	NA	1,373	1,012	892	151	98	33	2,276
P. falciparum	de novo k55	NA	NA	1,360	1,074	847	218	97	37	2,205
P. falciparum	de novo k61	NA	NA	1,330	980	898	147	98	36	2,226
P. falciparum	de novo k65	NA	NA	1,283	718	973	143	98	47	2,120
P. falciparum	de novo k71	NA	NA	968	550	1,288	97	99	37	1,488
P. falciparum	Version 2.1.4	NA	NA	1,445	730	712	176	97	72	2,189
P. falciparum	Version 3	NA	NA	1,445	1,058	705	232	97	157	2,192
C. elegans	WS228	NA	NA	6,301	1,206	121,864	1,734	17,898	209	14,969
M. musculus	GRCm38	6,060	1,026,7929	8,451	26,610	7,513,384	7,123	96,932	2,280	217,097
H. sapiens	GRCh37	5,327	927,5230	6,750	30,913	7,686,565	5,661	102,291	2,929	433,700

Table S7: Memory and running time of read mapping and REAPR. For all genomes except human and mouse, the short insert perfect and unique coverage was calculated using the REAPR function `perfectmap`. Due to their size, these data for the human and mouse genomes were generated by mapping the reads with SMALT, then using the REAPR function `perfectfrombam`. Therefore columns five and six refer to the resource usage of `perfectfrombam`, (not `perfectmap`) for the human and mouse genomes.

Scaffold	Reason	Type	Start	End	FCD error	True Positive	Comments
velvet.89	No fragment coverage	Scaffold	36,739	36,833	NA	1	scaffolding error
velvet.89	FCD	Contig	85701	91,316	0.52	1	deletion from assembly
velvet.89	FCD error	Scaffold	337,350	343,185	0.59	1	scaffolding error
velvet.89	FCD	Contig	430,401	436,872	0.59	1	deletion from assembly
velvet.89	FCD	Contig	476,817	479,096	0.19	0	subpopulation of reads with too short insert
velvet.89	FCD error	Scaffold	480,786	483,165	0.20	1	gap 480,088..480,160 should be 534 bases
velvet.89	FCD	Contig	485,765	490,960	0.30	1	deletion from assembly
velvet.89	FCD	Contig	719,113	725,564	0.59	1	false join in contig
velvet.89	No fragment coverage	Contig	722,391	722,398	NA	1	false join in contig
velvet.89	No fragment coverage	Scaffold	756,475	756,548	NA	1	scaffolding error
velvet.89	No fragment coverage	Scaffold	826,114	826,184	NA	1	scaffolding error
velvet.89	FCD error	Scaffold	939,610	947,065	0.21	1	Very messy region over gaps
velvet.89	FCD error	Scaffold	947,578	950,605	0.25	1	Very messy region over gaps
velvet.93	FCD error	Scaffold	93,674	102,841	0.31	1	local translocation
velvet.93	No fragment coverage	Scaffold	535,587	535,669	NA	1	scaffolding error
velvet.93	FCD	Contig	555,885	562,332	0.59	1	false join in contig
velvet.93	FCD	Contig	586,757	592,828	0.57	1	false join in contig
velvet.93	No fragment coverage	Scaffold	748,213	748,311	NA	1	scaffolding error
velvet.93	FCD error	Scaffold	753,902	762,333	0.54	1	multiple errors
velvet.115	FCD error	Scaffold	11,630	17,609	0.54	1	scaffolding error
velvet.115	FCD error	Scaffold	24,710	30,941	0.48	1	multiple errors
velvet.115	No fragment coverage	Scaffold	86,075	86,148	NA	1	scaffolding error
velvet.115	No fragment coverage	Scaffold	99,223	99,302	NA	1	scaffolding error
velvet.115	No fragment coverage	Scaffold	124,161	124,227	NA	1	scaffolding error
velvet.115	No fragment coverage	Scaffold	225,271	225,352	NA	1	scaffolding error
velvet.115	No fragment coverage	Scaffold	243,944	244,011	NA	1	scaffolding error
velvet.169	No fragment coverage	Scaffold	15,231	15,300	NA	1	scaffolding error
velvet.169	FCD error	Scaffold	21,922	28,297	0.41	1	multiple errors
velvet.169	No fragment coverage	Scaffold	32,135	32,219	NA	1	scaffolding error
velvet.169	No fragment coverage	Scaffold	140,184	140,257	NA	1	scaffolding error
velvet.169	FCD error	Scaffold	177,710	184,585	0.39	1	scaffolding error
velvet.169	FCD error	Scaffold	232,390	237,696	0.53	1	multiple errors

Table S8: Error calls made by REAPR in the GAGE Velvet assembly of *S. aureus*.

Scaffold	Reason	Type	Start	End	FCD error	True Positive	Comments
scaffold2	FCD error	Contig	92,695	92,944	0.58	0	
scaffold2	FCD error	Scaffold	95,877	96,249	0.61	0	Noise around gap due to repeats
scaffold2	FCD error	Scaffold	100,850	102,043	0.71	1	Repetitive badly assembled region
scaffold2	FCD error	Scaffold	402,240	402,798	0.68	1	Negative gap
scaffold2	FCD error	Contig	403,877	404,178	0.58	1	Multiple hits to elwhere in genome
scaffold4	FCD error	Contig	185,376	185,762	0.58	0	
scaffold101	FCD error	Scaffold	40,965	41,438	0.66	0	LHS of gap is a reapeat
scaffold11	FCD error	Scaffold	5,805	7,199	0.70	1	Scaffolding error
scaffold19	FCD error	Contig	38,616	38,986	0.59	0	Matches reference ok, error caused by matching elsewhere with a repeat
scaffold20	FCD error	Contig	30,767	31,064	0.60	0	
scaffold22	FCD error	Scaffold	10,882	11,435	0.67	1	Negative gap
scaffold22	FCD error	Scaffold	14,026	14,552	0.67	1	Negative gap
scaffold22	FCD error	Scaffold	15,333	16,557	0.68	1	Negative gap
scaffold23	FCD error	Scaffold	1,460	1,816	0.90	1	Gap should be 430bp long instead of 12bp. This region also matches another part of genome where again gap 430bp in reference
scaffold25	FCD error	Scaffold	3,708	4,006	0.60	1	Negative gap
scaffold25	FCD error	Scaffold	8,722	10,052	0.72	1	Negative gap
scaffold25	FCD error	Scaffold	10,172	10,827	0.69	1	Negative gap
scaffold27	FCD error	Scaffold	2,995	3,495	0.66	1	Negative gap
scaffold27	FCD error	Scaffold	5,279	5,811	0.67	1	Negative gap
scaffold32	FCD error	Scaffold	1,046	1,606	0.67	1	Negative gap
scaffold33	FCD error	Contig	986	1,777	0.67	0	
scaffold34	FCD error	Scaffold	506	1,053	0.66	1	Negative gap
scaffold35	FCD error	Scaffold	315	768	0.66	1	Negative gap
scaffold41	FCD error	Scaffold	3	835	0.78	1	Negative gap

Table S9: Error calls made by REAPR in the *de novo* assembly of *S. aureus* TW20 with a k-mer of 71.

Scaffold	Reason	Type	Start	End	FCD error	True Positive	Comments
scaffold1	FCD error	Scaffold	605,738	609,841	0.73	1	Negative gap
scaffold1	No fragment coverage	Scaffold	612,891	613,112	NA	1	Scaffolding error
scaffold2	FCD error	Scaffold	357,446	364,753	0.78	1	Scaffolding error
scaffold2	FCD error	Scaffold	530,381	537,283	0.80	1	Scaffolding error
scaffold2	No fragment coverage	Contig	533,360	533,584	NA	1	Scaffolding error
scaffold3	No fragment coverage	Scaffold	228,791	229,393	NA	1	Scaffolding error
scaffold3	FCD error	Scaffold	247,736	255,511	0.73	1	Scaffolding error
scaffold3	FCD error	Scaffold	470,180	478,648	0.73	1	Scaffolding error
scaffold4	FCD error	Scaffold	261,947	269,317	0.87	1	Scaffolding error
scaffold5	No fragment coverage	Scaffold	16,715	17,356	NA	1	Scaffolding error
scaffold5	FCD error	Scaffold	201,098	209,530	0.81	1	Scaffolding error
scaffold5	FCD error	Scaffold	453,287	458,164	0.73	1	Scaffolding error
scaffold5	No fragment coverage	Scaffold	676,154	676407	NA	1	Scaffolding error
scaffold6	FCD error	Scaffold	192,845	200,890	0.77	1	Scaffolding error
scaffold6	FCD error	Scaffold	597,917	605,404	0.64	1	Scaffolding error
scaffold6	No fragment coverage	Contig	600,296	601454	NA	0	
scaffold7	FCD error	Scaffold	547,103	553,357	0.78	1	Scaffolding error
scaffold8	FCD error	Scaffold	42,158	47,224	0.78	1	Scaffolding error
scaffold9	FCD error	Scaffold	514,451	518,626	0.66	1	Deletion from assembly
scaffold10	No fragment coverage	Scaffold	299,730	300,095	NA	1	Scaffolding error
scaffold11	No fragment coverage	Scaffold	436,472	436,798	NA	1	Scaffolding error
scaffold11	No fragment coverage	Scaffold	494,773	498,590	NA	1	Scaffolding error
scaffold12	No fragment coverage	Scaffold	466,434	466,847	NA	1	Scaffolding error
scaffold13	No fragment coverage	Scaffold	159,639	162,503	NA	1	Scaffolding error
scaffold13	FCD error	Scaffold	518,294	523,693	0.81	1	Scaffolding error
scaffold16	FCD error	Scaffold	450,893	463,645	0.86	1	Scaffolding error
scaffold16	No fragment coverage	Contig	455,792	455,846	NA	0	
scaffold17	FCD error	Scaffold	5294	10,369	0.90	1	Scaffolding error
scaffold17	No fragment coverage	Scaffold	20,160	20,528	NA	1	Scaffolding error
scaffold17	No fragment coverage	Scaffold	36,707	37,186	NA	1	Scaffolding error
scaffold17	No fragment coverage	Scaffold	269,939	270,290	NA	1	Scaffolding error
scaffold18	No fragment coverage	Scaffold	141,263	141,544	NA	1	Scaffolding error
scaffold18	No fragment coverage	Scaffold	209,978	218,678	NA	1	Scaffolding error
scaffold18	No fragment coverage	Scaffold	379,841	381,107	NA	1	Scaffolding error
scaffold19	FCD error	Scaffold	197,962	208,648	0.73	1	Scaffolding error
scaffold19	No fragment coverage	Scaffold	362,796	363,397	NA	1	Scaffolding error
scaffold19	FCD error	Scaffold	396,569	401,378	0.75	1	Scaffolding error
scaffold20	No fragment coverage	Scaffold	56,102	56406	NA	1	Scaffolding error
scaffold21	No fragment coverage	Scaffold	155,886	156,219	NA	1	Scaffolding error
scaffold21	No fragment coverage	Scaffold	382,895	391,172	NA	1	Scaffolding error
scaffold26	No fragment coverage	Scaffold	314,426	316,169	NA	1	Scaffolding error
scaffold27	FCD error	Scaffold	80,867	89,146	0.76	1	Scaffolding error
scaffold27	No fragment coverage	Contig	83,766	84,330	NA	0	
scaffold28	No fragment coverage	Scaffold	97,353	98,220	NA	1	Scaffolding error
scaffold31	No fragment coverage	Scaffold	69,477	69,704	NA	1	Scaffolding error
scaffold31	No fragment coverage	Scaffold	90,135	105,450	NA	1	Scaffolding error
scaffold31	FCD error	Scaffold	236,909	244,054	0.73	1	Scaffolding error
scaffold35	FCD error	Scaffold	86,789	94,384	0.81	1	Negative gap
scaffold35	No fragment coverage	Contig	91,114	91,525	NA	1	Scaffolding error
scaffold35	No fragment coverage	Scaffold	172,854	173,063	NA	1	Scaffolding error
scaffold36	No fragment coverage	Scaffold	125,374	125,809	NA	1	Scaffolding error
scaffold40	No fragment coverage	Scaffold	59,554	60,106	NA	1	Scaffolding error
scaffold40	FCD error	Scaffold	174,238	181,540	0.76	1	Scaffolding error
scaffold40	No fragment coverage	Contig	177,763	178,345	NA	0	
scaffold51	No fragment coverage	Scaffold	76,358	77,661	NA	1	Scaffolding error
scaffold52	No fragment coverage	Scaffold	33,059	33,256	NA	1	Scaffolding error
scaffold57	No fragment coverage	Scaffold	26,646	29,229	NA	1	Scaffolding error
scaffold61	No fragment coverage	Scaffold	29,733	30,250	NA	1	Scaffolding error
scaffold63	No fragment coverage	Scaffold	29,995	30,448	NA	1	Scaffolding error
scaffold66	No fragment coverage	Scaffold	24,564	24,768	NA	1	Scaffolding error
scaffold81	FCD error	Scaffold	1,541	8,524	0.86	0	Messy contig end, many gaps
scaffold89	FCD error	Scaffold	10,595	15,301	0.68	1	Scaffolding error

Table S10: Error calls made by REAPR in the *de novo* assembly with *k*-mer 55 of *P. falciparum*
